# What influences child feeding in the Northern Triangle? A mixed‐methods systematic review

**DOI:** 10.1111/mcn.13018

**Published:** 2020-05-26

**Authors:** Megan Deeney, Helen Harris‐Fry

**Affiliations:** ^1^ Faculty of Epidemiology and Population Health London School of Hygiene & Tropical Medicine London UK

**Keywords:** behaviour change, breastfeeding, child feeding, diets, El Salvador, Guatemala, Honduras, infant and child nutrition, infant feeding, nutrition, systematic review

## Abstract

Optimising child feeding behaviours could improve child health in Guatemala, Honduras and El Salvador, where undernutrition rates remain high. However, the design of interventions to improve child feeding behaviours is limited by piecemeal, theoretically underdeveloped evidence on factors that may influence these behaviours. Between July 2018 and January 2020, we systematically searched Cochrane, Medline, EMBASE, Global Health and LILACS databases, grey literature websites and reference lists, for evidence of region‐specific causes of child feeding behaviours and the effectiveness of related interventions and policies. The Behaviour Change Wheel was used as a framework to synthesise and map the resulting literature. We identified 2,905 records and included 68 relevant studies of mixed quality, published between 1964 and 2019. Most (*n* = 50) were quantitative, 15 were qualitative and three used mixed methods. A total of 39 studies described causes of child feeding behaviour; 29 evaluated interventions or policies. Frequently cited barriers to breastfeeding included mothers' beliefs and perceptions of colostrum and breast milk sufficiency; fears around child illness; and familial and societal pressures, particularly from paternal grandmothers. Child diets were influenced by similar beliefs and mothers' lack of money, time and control over household finances and decisions. Interventions (*n* = 22) primarily provided foods or supplements with education, resulting in mixed effects on breastfeeding and child diets. Policy evaluations (*n* = 7) showed positive and null effects on child feeding practices. We conclude that interventions should address context‐specific barriers to optimal feeding behaviours, use behaviour change theory to apply appropriate techniques and evaluate impact using robust research methods.

**Key messages**

- We lack high‐quality evidence explaining child feeding behaviours in the Northern Triangle, particularly in El Salvador.
- Existing evidence on child feeding behaviours in the Northern Triangle focuses mainly on mothers' beliefs about the health benefits of breast milk and other foods, the influence of husbands and grandmothers and physical constraints such as time and money.
- Future child feeding interventions in the Northern Triangle should explicitly use and build on existing evidence on context‐specific drivers of behaviour and use behaviour change theory at the formative design stages.

## 1 INTRODUCTION

Child undernutrition has serious health and economic effects at the individual, household and national levels (Victora et al., 2008). In Northern Triangle countries (Guatemala, Honduras and El Salvador), the cost of undernutrition is around 5 billion US dollars per year (ECLAC, 2008). Around 14% to 47% of children are short for their age in these countries, and, although undernutrition is in decline, the rate of change is slow, uneven and further complicated by rising levels of child overweight and obesity (Ministerio de Salud/Instituto Nacional de Salud & UNICEF, 2014; MSPAS, INE, & ICF, 2017; Secretaría de Salud, INE, & ICF, 2013).

Improved child feeding practices, including breastfeeding and complementary feeding, improve nutrition outcomes (Victora et al., 2016). However, in the Northern Triangle, current practices are suboptimal. Between one third to one half of children aged under 6 months are exclusively breastfed (PAHO & WHO, 2017), and only half of children aged 6–23 months receive the World Health Organization (WHO)‐defined ‘minimum acceptable diet’ (UNCF, 2018).

Furthermore, it remains unclear how to most effectively improve feeding practices. Globally, interventions have had large but heterogeneous effects on breastfeeding (Sinha et al., 2015) and smaller effects on complementary feeding and nutrition outcomes (Dewey & Adu‐Afarwuah, 2008). Within the Northern Triangle, various programmes and policies have been implemented, but little is known about their relative effectiveness.

Previous research on child feeding behaviours in the Northern Triangle has identified determinants such as socio‐economic status (Mock, Franklin, Bertrand, & O'Gara, 1985) and maternal education (Webb, Sellen, Ramakrishnan, & Martorell, 2009). However, this can only guide interventions towards population subgroups, rather than explain the causes of behaviour (O'gara & Kendall, 1985).

Moreover, evidence on determinants, interventions and policies to improve child feeding has typically ignored behaviour change theory (Webb Girard, Waugh, Sawyer, Golding, & Ramakrishnan, 2019). Theory can help decipher the causes of behaviour and identify appropriate behaviour change techniques (Briscoe & Aboud, 2012). Theory‐based interventions can iteratively inform theory and help to unpack why interventions succeed or fail (Michie, van Stralen, & West, 2011).

**FIGURE 1:**
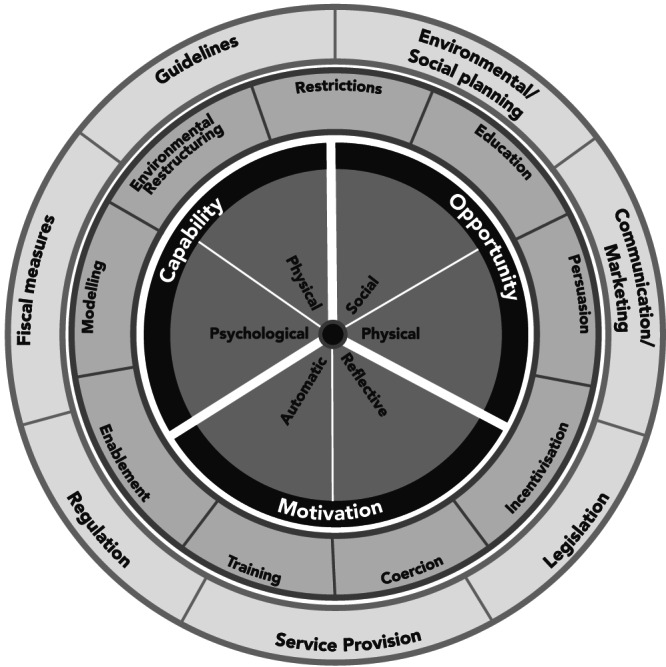
Behaviour Change Wheel by Michie, Van Stralen, and West. Notes: Recreated by authors, based on the original (Michie et al., 2011)

One model for understanding behaviour is the ‘Behaviour Change Wheel’—the product of a systematic review and synthesis of previous behaviour change frameworks (Figure 1) (Michie et al., 2011).

At the centre of the framework is the capabilities, opportunities, motivations and behaviour (‘COM‐B') system, which explains why behaviours are adopted:

- ‘Capabilities’ are the psychological and physical ability to behave in a certain way, for example, problems with latching.
- ‘Motivations’ are the automatic and reflective brain processes that drive behaviours, like children's tastes and caregivers' desires to have healthy children.
- ‘Opportunities’ are social and physical external factors that influence behaviour, like social support for breastfeeding or food availability (Michie et al., 2011; Michie, Atkins, & West, 2018).

These determinants can act singularly or in combination to influence behaviour. Circling the COM‐B core are categories of interventions and policies that influence capabilities, opportunities, motivations and the behaviour of interest (Michie et al., 2018, 2011).

We systematically review evidence on the capabilities, motivations and opportunities that determine infant and young child feeding (IYCF) behaviours, alongside evidence for the effectiveness of interventions and policies that address these behaviours in the Northern Triangle. Using the Behaviour Change Wheel to map the evidence, we identify gaps in the understanding of child feeding behaviours.

## 2 METHODS

We followed the Preferred Reporting Items for Systematic Reviews and Meta‐Analyses (PRISMA) reporting guidelines (Moher et al., 2009). The study protocol was developed in consultation with librarians from the London School of Hygiene & Tropical Medicine and prospectively registered (Deeney & Harris‐fry, 2018).

### 2.1 Search strategy

Between July 2018 and March 2019, we searched five databases, grey literature websites and reference lists. Databases were Cochrane Database of Systematic Reviews (1999 to present), Medline (1950 to present), EMBASE (1980 to present), Global Health (1973 to present) and LILACS (1982 to present). Websites were governments, Ministries of Health, the Emergency Nutrition Network Field Exchange, WHO, the Food and Agriculture Organization, United Nations Children's Fund, the World Food Programme, Save the Children and Oxfam. Based on reviewer feedback, in January 2020, we searched FHI 360, The Manoff Group, the Inter‐American Development Bank's Salud Mesoamérica Initiative and USAID Food and Nutrition Technical Assistance Project (FANTA).

Our search string (dollar sign, $, indicating a truncation) was as follows:

(breastfeed$ or breast‐feed or breastfed or breast‐fed or “breast fed” or lacta$ or colostrum or “bottle feed” or bottle‐feed or bottle‐fed or wean$ or feeding or nutrition or “solid food” or “semi‐solid food” or “soft food” or diet$ or meal or formula) AND (child$ or infant$ or baby or babies) AND (guatemala or honduras or “el salvador” or “northern triangle”).

Equivalent Spanish search terms were applied to LILACS database and grey literature websites.

### 2.2 Inclusion criteria

We included all relevant quantitative (experimental or observational), qualitative, peer‐reviewed and grey literature, with no publication date limits. The study population comprised children less than 5 years of age and their parents or caregivers in Guatemala, Honduras or El Salvador. Exposures were the capabilities, motivations and opportunities that directly influence IYCF behaviours, or an intervention or policy that targeted IYCF behaviours. Outcomes were any measure or description of IYCF.

Literature was excluded if it did not report original evidence, or only characterised feeding behaviours.

### 2.3 Data screening and extraction

Search results were imported into EndNote reference management software. Two authors independently screened the results, and disagreements were resolved by discussion. We extracted the following prespecified data: author, publication date, study design, location, population, sample size, exposure (source of behaviour, intervention or policy type), outcome measure, analysis method and key findings.

### 2.4 Quality assessment

We used an adapted version of the Risk of Bias in Non‐Randomised Studies of Interventions (ROBINS‐I) tool (Sterne et al., 2016) to assess the risk of bias. The Critical Appraisal Skills Programme (CASP) checklist (CASP, 2018) was used to assess qualitative results. To map the evidence, we categorised CASP scores as high (≥8), moderate (5 to 7) or low (<5) quality.

### 2.5 Analysis

Two reviewers independently mapped exposures onto the Behaviour Change Wheel, and disparities in categorisation were resolved through discussion. As we anticipated high heterogeneity in exposure–outcome pairings, we did not plan to conduct a meta‐analysis.

## 3 RESULTS

### 3.1 Study characteristics

**FIGURE 2:**
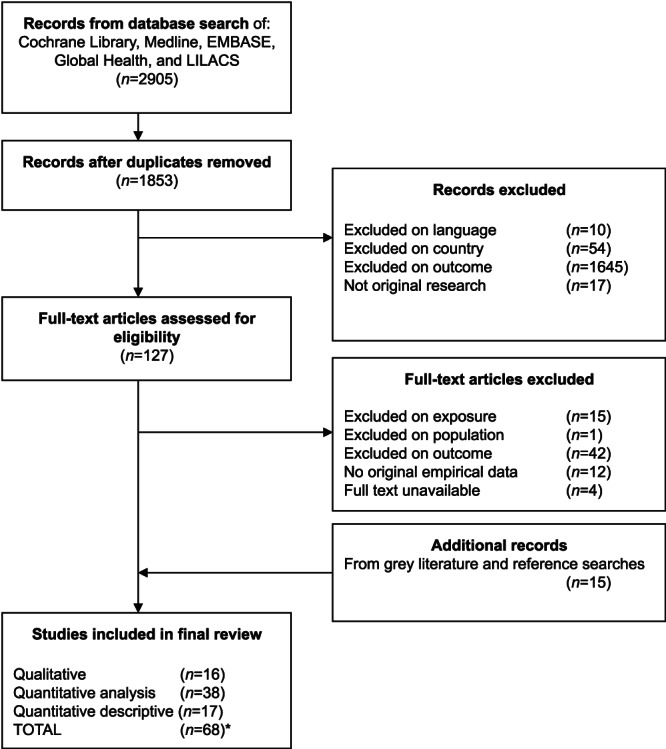
Flow diagram of study selection. Notes: Final numbers add up to 71 because three studies used mixed methods

Database searches returned 1,853 unique results with an additional 15 records from searching websites and reference lists. Of these, 68 were included in our review (Figure 2).

Characteristics of studies on the sources of behaviour are given in Table 1 and on interventions and policies in Table 2 **.**

**TABLE 1:** Study characteristics of evidence for the influences on child feeding in the Northern Triangle and assigned ‘COM’ categories

Author, year	Study design (data collection method)	Sample size (study population)	Analysis method	Capability		Opportunity		Motivation		Outcome
				Phy	Psy	Soc	Phy	Aut	Ref	
**Guatemala (*n* = 30)**										
Atyeo, Frank, Vail, Sperduto, & Boyd, 2017	Cross section (interviews)	*n* = 178 mothers of any age (indigenous, rural)	Framework coding and chi‐squared tests	✗	✓	✓	✓	✗	✓	EIBF (G)
Brown et al., 2016	Cross section (FGDs)	*n* = 101 caregivers of children 6–36 months (indigenous, rural)	Thematic coding	✗	✗	✓	✓	✗	✓	Child feeding (G)
Chary, Messmer, & Rohloff, 2011	Case studies (observations and interviews)	*n* = 3 households with children <2 years (indigenous, rural)	Not stated	✗	✗	✓	✗	✗	✗	Child feeding (G)
Dearden, Altaye, de Maza, de Oliva, Stone‐Jimenez, Morrow, et al., 2002	Preintervention baseline cross section (interviews)	*n* = 777 households with children <6 months (mixed ethnicity, peri‐urban)	Chi‐squared tests and logistic regression	✗	✓	✓	✓	✗	✗	EIBF and EBF
Engle & Nieves, 1992	Cross section (observations and interviews) of mothers	*n* = 45 mothers who had one child 1–5 years (Ladino, peri‐urban, in supplementation programme)	*t* tests	✗	✓	✗	✗	✗	✗	Child feeding (G)
Garcia, Padilla, Doak, Vossenaar, & Solomons, 2012	Cross section (semistructured interviews and FGDs)	*n* = not specified, mothers of children <24 months (indigenous and European, urban)	Qualitative synthesis	✗	✗	✓	✗	✗	✓	Child feeding (G)
Garcia‐Meza et al., 2017	Cross section (interviews)	*n* = 9 mothers who had three children each, between 6–8, 9–11 and 12–24 months (urban)	Thematic coding	✗	✓	✗	✗	✓	✓	Feeding micronutrient powders and supplements
Garcia‐Meza, Montenegro‐Bethancourt, et al., 2017	Cross section (interviews)	*n* = 9 mothers of children 0–23 months (urban)	Not stated	✗	✗	✓	✓	✗	✓	Delayed complementary feeding
Gonzalez et al., 2017	Non‐randomised trial with qualitative process evaluation (interviews and FGDs)	*n* = 28 caregivers of children 6–24 months (urban)	Not stated	✗	✗	✓	✓	✓	✓	Use of home‐fortification product
Grajeda & Perez‐Escamilla, 2002	Longitudinal cohort (clinical assessments)	*n* = 136 mothers (urban, low income)	Chi‐squared and Student's *t* tests, analysis of variance	✓	✓	✗	✗	✗	✗	Delayed onset of lactation >3 days postpartum vs. ≤3 days
Hruschka, Sellen, Stein, & Martorell, 2003	Longitudinal cohort (interviews)	*n* = 328 mothers followed up for 6 months postpartum (rural)	Cox proportional hazards regression	✓	✓	✗	✗	✗	✗	Risk of ending breastfeeding <6 months
Immink & Alarcon, 1993	Two cross sections	*n* = 786 households with one child 12–60 months if present (rural)	Probit regression	✗	✗	✗	✓	✗	✗	Household and preschooler dietary energy intake (kcal/day)
Izurieta & Larson‐Brown, 1995	Cross section (surveys and participant observation)	*n* = 65 families of children <5 years (rural)	Not stated	✗	✗	✓	✗	✗	✓	Introduction of complementary foods and meal frequency (G)
Kincaid et al., 2013	Cross section (semistructured interviews and FGDs)	*n* = 18 pregnant and early lactating women (indigenous, rural)	Thematic coding	✗	✗	✓	✗	✗	✓	Child feeding (G)
Martorell, Yarbrough, Yarbrough, & Klein, 1980	Longitudinal cohort (interviews)	*n =* 477 children 15–60 months and their mothers (rural)	Analysis of variance	✓	✗	✗	✗	✗	✗	Energy intake (kcal/day) protein intake (g/day)
Mata, Kromal, Urrutia, & Garcia, 1977	Prospective cohort (not specified: likely observation and interviews)	*n* = 30 weaned children <3 years (rural)	Correlation	✓	✗	✗	✗	✗	✗	% of recommended energy and protein intake after weaning
Matias, Chaparro, Perez‐Exposito, Peerson, & Dewey, 2011	Randomised crossover trial (interviews)	*n* = 42 children aged 6–18 months and their caregivers (mostly indigenous, rural)	Linear regression, chi‐squared and Fisher's tests	✓	✗	✗	✗	✓	✗	% of supplement consumed, caregivers' perceptions of supplement
McKerracher, Collard, Altman, Sellen, & Nepomnaschy, 2016	Cross section (interviews)	*n* = 51 mothers and 283 children (indigenous, rural)	Linear regression	✗	✗	✓	✓	✓	✓	Months of breastfeeding
Newman et al., 2014	Process evaluation of a complementary feeding trial (interviews)	*n* = 219 caregivers (semirural)	Chi‐squared tests	✓	✗	✗	✓	✓	✗	Days of child not consuming particular foods
Olney et al., 2012	Formative research for (Programa Comunitario Materno Infantil de Diversificación Alimentaria) using mixed‐methods cross‐sectional approach including semistructured interviews and FGDs	Semistructured interviews: *n* = 30 pregnant women and mothers of children aged 0–23 months, *n* = 10 fathers and *n* = 10 grandmothers of children aged 0–23 months FGDs: *n* = 5 pregnant women and mothers of children aged 0–23 months, *n* = 5 fathers and *n* = 5 grandmothers of children aged 0–23 months Acceptability of LNS and MNP: *n* = 50 children aged 6–23 months and *n* = 49 pregnant women or with children aged <6 months; *n* = 48 children aged 6–23 months and *n* = 48 pregnant women or with children aged 0–5 months, respectively (peri‐urban and rural)	Thematic coding	✓	✓	✓	✓	✓	✓	Acceptability of LNS and MNP, breastfeeding and complementary feeding
Parker, Schroeder, Begin, & Hurtado, 1998	Cross section (interviews and FGDs)	*n* = 46 mothers of children 6–14 months (indigenous, rural)	Descriptive statistics	✓	✗	✗	✗	✗	✓	Views on consistency of complementary foods
Pigott & Kolasa, 1979	Cross section (interviews)	*n* = 62 preschool children (1–6 years) (Ladino, rural)	Not stated	✗	✗	✓	✓	✗	✓	Child feeding (G)
Solien De Gonzalez, 1964	Cross section (questionnaires and interviews)	*n =* 57 families (Ladino, ‘lower class’, urban)	Not stated	✗	✗	✓	✗	✗	✓	Child feeding (G)
Tumilowicz, Habicht, Pelto, & Pelletier, 2015	Cross section (interviews)	*n* = 24 mothers of children 0–35 months (indigenous, rural)	Thematic coding	✗	✗	✓	✗	✗	✓	Child feeding (G)
Vemury & CARE, 1981	Cross section (interviews)	*n* = 400 mothers of preschool children 0–6 years (rural)	Summary statistics	✗	✗	✓	✓	✗	✓	Child feeding (G)
Vossenaar et al., 2012	Cross section (interviews)	*n* = 300 mothers of children 6–23 months (highlands)	Summary statistics	✗	✗	✓	✗	✗	✓	Child feeding (G)
Vossenaar, Garcia, Solomons, et al., 2012	Cross section (interviews and FGDs)	*n* = 49 mothers of children 6–23 months (indigenous and Ladino, urban highlands)	Thematic coding	✗	✗	✓	✗	✗	✓	Introduction of agüitas (liquids given to children) (G)
Wehr, Chary, Webb, & Rohloff, 2014	Cross section (FGDs)	*n* = 51 caregivers of children aged 6–36 months (indigenous/rural)	Thematic coding	✗	✗	✓	✓	✗	✓	Child feeding (G)
World Health Organization, 1981	Cross section (interviews and questionnaires)	*n* = 1,785 mother–child pairs (economically advantaged, urban poor and rural)	Descriptive statistics	✗	✗	✗	✓	✗	✓	Child feeding (G)
Wren, Solomons, Chomat, Scott, & Koski, 2015	Cross section (questionnaires)	*n* = 190 mother–child aged <46 days dyads (indigenous, rural)	Logistic and linear regression	✗	✗	✓	✗	✗	✓	EIBF
**Guatemala and El Salvador (*n* = 1)**										
Nieves et al., 1994	Cross section (interviews and FGDs)	*n* = 64 Guatemalan women and *n* = 73 El Salvadorian women (rural)	Not stated	✗	✗	✓	✓	✗	✓	Child feeding (G)
**El Salvador (*n* = 1)**										
Cerezo & Claros, 1993	Cross section (interviews)	*n* = 128 new mothers (no description given)	Summary statistics	✗	✓	✓	✗	✗	✓	Breastfeeding (G)
**Honduras (*n* = 7)**										
Cohen, Rivera, Canahuati, Brown, & Dewey, 1995	Prospective cohort (interviews)	*n* = 63 multiparous women (no description given)	Summary statistics	✗	✗	✓	✓	✗	✓	Interruption of EBF <6 months (G)
Cohen, Brown, Rivera, & Dewey, 1999	Cross section (FGDs)	*n* = 5 focus groups of 6–8 women (peri‐urban)	Summary statistics	✗	✗	✓	✗	✗	✓	EBF (G)
Gutiérrez Cabrera & Turcios España, 2004	Cross section (interviews)	*n* = 177 mothers of children <2 years (urban)	Summary statistics	✗	✓	✗	✗	✗	✗	Any breastfeeding at the time of interview
O'gara & Kendall, 1985	Ethnographic prospective cohort (interviews)	*n* = 75 mothers and families with newborn children (low income/peri‐urban)	Not stated	✗	✓	✓	✗	✗	✓	Perceptions of breast milk, breastfeeding and substitute milks (G)
Lutter et al., 1994	Prospective cohort (hospital records and interviews)	*n* = 1,582 women recruited at maternity wards (urban hospitals)	Chi‐squared tests and one‐way analysis of variance	✗	✗	✓	✓	✗	✓	EBF
Perez‐Escamilla et al., 1995	Prospective cohort (interviews)	*n* = 873 mothers followed from birth of child to 4 months (urban, low income)	Chi‐squared tests and Cox regression	✗	✗	✗	✗	✗	✓	Probability of EBF at 1 and 2 months EBF duration
Perez‐Escamilla, Segura‐Millan, Canahuati, & Allen, 1996	*Encuesta Nacional De Epidemiología y Salud Familiar* national survey	*n* = 714 children 0–6 months (nationally representative)	Logistic regression	✓	✓	✗	✗	✗	✗	EBF Any breastfeeding

Abbreviations: Aut, automatic; COM, capabilities, opportunities and motivations; EBF, exclusive breastfeeding; EIBF, early initiation of breastfeeding (within 1 h postpartum); FGD, focus group discussion; G, general topic of discussion; Phy, physical; Psy, psychological; Ref, reflective; Soc, social.

**TABLE 2:** Study characteristics of evidence for the interventions and policies for child feeding in the Northern Triangle and assigned Behaviour Change Wheel categories

Author (date)	Study design and description of activities	Sample size (study population)	Analysis method	Behaviour Change Wheel intervention function	Outcome measure
**Interventions**					
**Guatemala *n* = 14**					
Asensio, 2013	Cluster control trial: intervention participants in one township received antiparasitic medication, multivitamins, educational messages and home‐prepared recipes. Another township acted as control	*n* = 2 clusters; 51 children aged 0–5 years in intervention cluster (rural)	*t* tests	Education	Child feeding behaviours (unspecified)
Dearden, Altaye, de Maza, de Oliva, Stone‐Jimenez, Burkhalter, et al., 2002	Programme impact evaluation using repeated cross‐sectional surveys: *La Leche League*'s breastfeeding promotion programme providing counsellor‐facilitated mother‐to‐mother support groups	*n* = 768 children <6 months in two programmes and two control communities (low income, peri‐urban)	Logistic regression	Education, persuasion, training	EIBF EBF
Gonzalez‐Cossio, Habicht, Rasmussen, & Delgado, 1998	Randomised controlled trial: intervention was a high‐energy supplement, controls received a placebo	*n* = 102 lactating women (mainly indigenous, rural)	Analysis of variance and linear regression	Enablement	EBF
Health and Development Consulting International (HDCi) LLC, 2013	Programa Comunitario Materno Infantil de Diversificación Alimentaria (PROCOMIDA) All intervention groups received BCC and different arms received different family food rations: full family ration (FFR), reduced family ration (RFR) or no family ration (NFR) and different individual rations: corn–soy blend (CSB), lipid‐based nutrient supplement (LNS), micronutrient powder (MNP), compared with a control group that received neither BCC nor food rations Midterm compared with baseline, using repeated cross‐sectional surveys	*n* = 942 households with children aged 0–59 months (predominantly indigenous, rural)	*t* test and chi‐squared test	Enablement, education, incentivisation	EBF, % of children aged 6–24 months with minimum acceptable dietary diversity
Heckert, Leroy, Bliznashka, Olney, & Richter, 2018	Programa Comunitario Materno Infantil de Diversificación Alimentaria (PROCOMIDA) All intervention groups received BCC and different arms received different family food rations: full family ration (FFR), reduced family ration (RFR) or no family ration (NFR) and different individual rations: corn–soy blend (CSB), lipid‐based nutrient supplement (LNS), micronutrient powder (MNP), compared with a control group that received neither BCC nor food rations Impact evaluation using longitudinal cohort household surveys	*n* = between 3,926 and 4,194 children aged ≤24 months (predominantly indigenous, rural)	Summary statistics, joint *f* test, Pearson chi‐squared test, fixed effect model	Enablement, education, incentivisation	EIBF, EBF, predominant breastfeeding, % children breastfed in the last 24 h, consumption of semi solid foods, minimum meal frequency, number of food groups consumed in last 24 h, minimum dietary diversity, consumption of iron‐rich or iron‐fortified foods, minimum acceptable diet
Islam & Hoddinott, 2009	Secondary data analysis of a cluster‐randomised controlled trial: intervention participants received Atole (a high‐protein energy drink). Controls got a low‐calorie drink.	*n* = 8,332 children 0–7 years^a^ (mixed ethnicities, rural)	Multiple regression	Enablement	Daily energy intake Daily energy intake at home Daily energy intake from Atole
Kennedy, 1994	Comparative analysis of case studies: *Cuatro Pinos* farmers cooperative adopting export vegetable production	*n* = 383 households (predominantly indigenous, rural)	None: summary data presented	Enablement	Age weaned (months)
Krebs et al., 2012	Cluster‐randomised trial: provision of meat, compared with equicaloric micronutrient‐fortified rice–soy cereal product	*n* = 300 to 329 children aged 3–4 months (semirural)	Logistic regression and Fisher's exact tests	Enablement and education	% mothers breastfeeding and bottle feeding No. of main meals per day and additional meals per day No. of food groups consumed
Martinez et al., 2018	Randomised controlled trial: delivered to mother–child dyads assigned to receive either standard care, consisting of generic age‐based complementary feeding messages delivered by community health workers (control), or the intervention consisting of standard care plus individualised complementary feeding education delivered through structured interviews, 24‐h dietary recalls and open‐ended goal‐setting questions	*n* = 324 children aged 6–24 months with a height‐for‐age *Z* score of ≤−2.5 *SD* (indigenous, rural)	Risk ratios (RR) and 95% confidence intervals	Education	Minimum dietary diversity, minimal acceptable diet, minimum meal frequency
Martorell et al., 1979	Before and after study: provision of food ration (100‐g beans and 90‐g corn per day per individual)	*n* = 40 children <6 years (rural)	Paired *t* test	Enablement	Daily energy (kcal) and protein (g) intake
Olney et al., 2013	Programa Comunitario Materno Infantil de Diversificación Alimentaria (PROCOMIDA) All intervention groups received BCC and different arms received different family food rations: full family ration (FFR), reduced family ration (RFR) or no family ration (NFR) and different individual rations: corn–soy blend (CSB), lipid‐based nutrient supplement (LNS), micronutrient powder (MNP), compared with a control group that received neither BCC nor food rations process evaluation using cross‐sectional design with random sampling, mixed methods	*n* = 108 pregnant women or with a child aged ≤24 months (predominantly indigenous, rural)	Summary statistics	Enablement, education, incentivisation	Child diets (G), minimum dietary diversity, EIBF, introduction of liquids other than breast milk before 6 months, delayed introduction of complementary foods and feeding practices during illness
Olney, Leroy, Bliznashka, & Ruel, 2018	Programa Comunitario Materno Infantil de Diversificación Alimentaria (PROCOMIDA) All intervention groups received BCC and different arms received different family food rations: full family ration (FFR), reduced family ration (RFR) or no family ration (NFR) and different individual rations: corn–soy blend (CSB), lipid‐based nutrient supplement (LNS), micronutrient powder (MNP), compared with a control group that received neither BCC nor food rations Impact evaluation using cluster‐randomised controlled trial	*n* = 3,404 children aged ≤24 months (predominantly indigenous, rural)	Linear mixed models and simple effects tests	Enablement, education, incentivisation	Use of CSB, LNS or MNP in the last 24 h (yes/no) and frequency of use in the last week
Sosa, Kennell, Klaus, & Urrutia, 1976	Randomised controlled trial: intervention mothers were left alone with their newborn for 45 mins and encouraged to breastfeed. Control participants were separated from their child until 12 h postpartum	*n* = 160 mother–newborn dyads (urban)	Unstated	Enablement, environmental restructuring	Duration of breastfeeding during the first year (mean no. of days)
Valverde et al., 1979	Before and after study: participants were given two high‐energy cookies daily for 4 weeks	*n* = 37 children <5 years (predominantly indigenous, rural)	Paired *t* test	Enablement	Daily intakes of energy (kcal), protein (g) and specific food items
**Honduras *n* = 7**					
Cohen, Brown, Canahuati, Rivera, & Dewey, 1994	Three‐arm randomised trial: (1) EBF vs. (2) introduction of complementary foods at 4 months, with ad libitum nursing 4–6 months, vs. (3) introduction of complementary foods at 4 months, with maintenance of baseline nursing frequency 4–6 months. Mothers were provided with complementary foods.	*n* = 141 mothers with newborns (low income)	Analysis of variance and chi‐squared tests	Enablement, education	Nursing frequency (times/day) nursing duration (min/day) breast milk intake (g/day) energy intake (kcal/day)
Cohen, Rivera, et al., 1995	Three‐arm randomised trial: (1) EBF vs. (2) introduction of complementary foods at 4 months, with ad libitum nursing 4–6 months, vs. (3) introduction of complementary foods at 4 months, with maintenance of baseline nursing frequency 4–6 months. Mothers were provided with complementary foods.	*n* = 141 mothers with newborns (low income)	Analysis of variance and Fisher's *t* test	Enablement, education	EBF and food intake (g) Food acceptance score: 1) ‘eats well’, 2) ‘accepts’, 3) ‘difficult to get child to eat the food’ and 4) ‘refuses’.
Dewey, Cohen, Brown, & Rivera, 1999	Two‐arm randomised trial: both groups received encouragement and motivational messages about breastfeeding. EBF group instructed to continue EBF to 6 months, SF group given complementary foods from 4 months to feed two times per day alongside normal breastfeeding	*n* = 119 mothers of low birthweight term children	Student's *t* test, chi‐squared tests and analysis of variance	Enablement, education, persuasion	Nursing frequency (times/day) Nursing duration (min/day) Breast‐milk intake (g/day) Energy intake (kcal/day)
Flax, Siega‐Riz, Reinhart, & Bentley, 2015	Cluster‐randomised controlled trial: participants in both groups received food vouchers and monthly nutrition education. Intervention group also received Plumpy'doz (lipid‐based nutrient supplement)	*n* = 298 children 6–18 months and caregivers (no description)	Linear regression	Enablement, education	Total energy (kcal), vitamin A retinol equivalents (μg), iron (mg)
Horton et al., 1996	Secondary data analysis of a cohort study: breastfeeding promotion programmes conducted at maternity services including education and support	*n* = 389 children aged 2 months (low income)	Summary statistics	Education	% children not breastfed, partially breastfed and EBF
Lutter, Pérez‐Escamilla, Segall, Sanghvi, Teruya, & Rivera, 1994	Impact evaluation using prospective cohort study: baby‐friendly hospital initiative‐related activities	*n* = 1,582 women recruited at maternity wards (urban hospitals)	Chi‐squared tests and survival analysis	Education, modelling, restrictions	EBF
Smith, 2002	Repeated cross‐section quasi‐experiment: Project HOPE's "Village Health Banks" programme of credit only banks with health education vs. without health education	*n* = 981 women (low income, rural)	Probit regression	Enablement, education	Any breastfeeding at point of time of survey
**El Salvador *n* = 1**					
Pérez‐Escamilla, 2004	Repeated cross section at baseline and follow‐up: Training of health care workers and information management system to improve breastfeeding counselling.	***n* = 1,457 to 3,004 women**	Chi‐squared tests and analysis of variance	Training, environmental restructuring	Any breastfeeding in the delivery room Any breastfeeding during first 30 min postpartum
**Policy evaluations**					
**Guatemala *n* = 1**					
Grajeda & Campos, 2016	Mixed methods evaluation of document analysis and cross‐sectional survey: *La Estrategia de Comunicación para el Desarrollo en Seguridad Alimentaria y Nutricional:* promoting child feeding practices using communications including sociodramas, community nutrition lotteries, TV and radio programmes	*n* = 88 families (mainly indigenous, urban and rural)	Thematic coding and narrative description	Communication/marketing	EBF complementary feeding (G)
**Honduras *n* = 6**					
American Public Health Association, 1987	Pilot study evaluation using repeated cross sections *Proyecto de Apoyo a la Lactancia Materna* (PROALMA). Promoting breastfeeding practices by training health care professionals to provide counselling to women and changing hospital policies between 1982 and 1988	*n* = 912 women in 1982 and *n* = 535 women in 1985 in the same 19 neighbourhoods	Summary statistics	Regulation, guidelines and service provision	Initiation of breastfeeding Average age of stopping breastfeeding % children breastfed at 12 months Average age of introduction of supplementary bottles % women introducing supplementary foods at 1 month
Canahuati, 1990	Extension activities evaluation using nonrandomised trial (talks + postnatal appointments) *Proyecto de Apoyo a la Lactancia Materna* (PROALMA). Promoting breastfeeding practices by training health care professionals to provide counselling to women and changing hospital policies between 1982 and 1988	*n* = 668 women	Not reported	Regulation, guidelines, service provision	Prevalence of breastfeeding at 6 months Prevalence of EBF at 90 days
Popkin, Canahuati, Bailey, & O'Gara, 1991	Programme evaluation with repeated cross section: *Proyecto de Apoyo a la Lactancia Materna* (PROALMA). Promoting breastfeeding practices by training health care professionals to provide counselling to women and changing hospital policies between 1982 and 1988	*n* = 1,540 to 3,354 children <24 months (nationally representative)	Trend analysis	Regulation, guidelines, service provision	Probability of initiating breastfeeding
Schaetzel, Griffiths, Miller Del Rosso, & Plowman, 2008	Atención integral a la Niñez en la Comunidad (AIN‐C) programme led by the Ministry of Health delivering growth monitoring and promotion activities through community volunteers Final report: original evaluation plan for before and after testing of designated AIN‐C and control communities was not possible due to a failure of community matching and extensive contamination of control communities with AIN‐C activities. A cross section of participants was therefore randomly sampled (from 92 communities) based on individual‐level participation in programme activities (AIN) vs. no exposure to growth monitoring and promotion (no GMP)	*n* = 1,213 children aged 0–23 months and their caregivers (not clustered) (rural)	Pearson, chi‐squared test and analysis of variance (ANOVA) logistic regression	Service provision	Feeding during illness (proportion who increase, maintain, decrease, stop), EBF, appropriate feeding for age group
Sierra, Espinoza, Espinoza, Espinoza, & Espinoza, 2019	AIN‐C (Atención Integral a la Niñez en la Comunidad) programme led by the Ministry of Health delivering growth monitoring and promotion activities through community volunteers: special project of AIN‐C implemented by decentralised providers in 1,038 rural communities launched in 2008 Quasi‐experiment using repeated surveys of intervention and control communities	2010 (baseline): *n* = 2,861 children <2 years, 2011: *n* = 3,152; 2012: *n* = 2,938 children <2 years (rural)	Not stated	Service provision	EBF
Van Roekel et al., 2002	AIN‐C (Atención Integral a la Niñez en la Comunidad) programme led by the Ministry of Health delivering growth monitoring and promotion activities through community volunteers Midterm evaluation using community‐level longitudinal approach by randomly sampling individuals from subset of same community clusters as in baseline	*n* = 1,168 children aged 0–23 months and their caregivers in *n* = 31 AIN‐C communities and *n* = 29 control communities (rural)	Pearson, chi‐squared test and analysis of variance (ANOVA) logistic regression	Service provision	Ever breastfed, currently breastfeeding, daily breastfeeding, EBF, introduction of liquids other than breast milk, child feeding scores based on frequency of breastfeeding, frequency of consumption of semisolid foods

Abbreviations: BCC, behaviour change communication; EBF, exclusive breastfeeding; EIBF, early introduction of breastfeeding; IYCF, infant and young child feeding; SF, group of children given supplementary food at 4 months.

a Children 6–7 years old were not eligible for our review though sample size by age group was not reported.

Studies were published between 1964 and 2019. Most were conducted in Guatemala (68%; *n* = 46), followed by Honduras (29%; *n* = 20), with very few in El Salvador (4%; *n* = 3) (one was in Guatemala and El Salvador). A total of 39 studies explored reasons behind child feeding behaviours; 22 described interventions, of which 10 were randomised trials; and seven were policy evaluations. Sample sizes ranged from three households to 8,332 children. Over half (56%) provided quantitative evidence of associations between at least one exposure and outcome, another 25% included no statistical analysis and 24% were qualitative. There were more studies on breastfeeding (*n* = 33) than on complementary feeding (*n* = 23), and 12 studies explored both.

### 3.2 Quality assessment

Risk of bias in quantitative studies (excluding studies containing only descriptive analyses) is summarised in Table S1. Only four studies were considered to be at low risk of bias, eight were ‘moderate’, and 26 were ‘serious’ or ‘critical’. Issues arose predominantly from the risk of confounding, missing data and participants not receiving their assigned intervention.

Quality of qualitative studies is summarised in Table S2. A common issue was a lack of reporting on recruitment strategy, participant–researcher relationship and analysis methods.

### 3.3 Mapping the evidence

Tables 1 and 2 describe and categorise evidence on the sources (Table 1) and interventions and policies (Table 2) that influence IYCF behaviours**.** Study results are given in Tables S3 (sources of behaviour) and S4 (interventions and policies).

**FIGURE 3:**
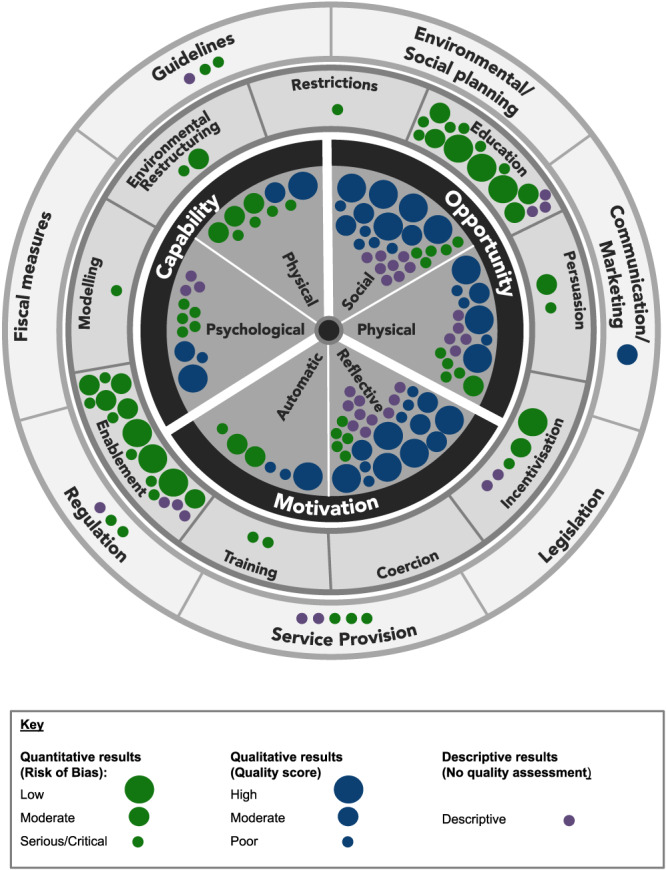
Evidence on determinants of child feeding behaviours, mapped onto the Behaviour Change Wheel by quality of evidence. Notes: Largest dots = high quality/low risk of bias; medium dots = moderate quality/moderate risk of bias; smallest dots = low quality/serious or critical risk of bias. Green = quantitative evidence (tests associations); purple = quantitative evidence (descriptive only); blue = qualitative evidence

**FIGURE 4:**
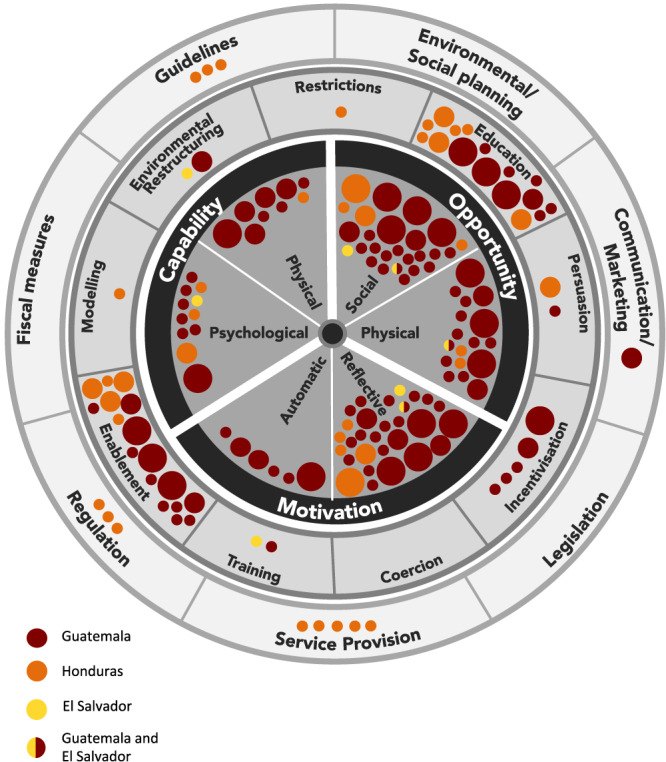
Evidence on determinants of child feeding behaviours, mapped onto the Behaviour Change Wheel by country of study. Notes: Largest dots = high quality/low risk of bias; medium dots = moderate quality/moderate risk of bias; smallest dots = low quality/serious or critical risk of bias. Red = Guatemala; orange = Honduras; yellow = El Salvador

Evidence is also mapped visually against the Behaviour Change Wheel and coded by data type (Figure 3) and study country (Figure 4). Many studies identified multiple exposures; each exposure is mapped individually.

### 3.4 Sources of IYCF behaviours

From 39 studies on the sources of IYCF behaviour, we identified and mapped 98 exposures to all six Behaviour Change Wheel subcategories.

#### 3.4.1 Reflective and automatic motivations

Reflective motivations were most frequently explored (28 studies). Studies had wide‐ranging publication dates (1979 to 2017) but consistent conclusions.

We found dichotomised views on colostrum—some mothers believed that it ‘cleans the stomach’, whereas others viewed it as bad or causing illness (Atyeo et al., 2017). Negative beliefs were reported in older and more recent studies (Atyeo et al., 2017; O'gara & Kendall, 1985). Mothers who did not believe that breast milk could transmit negative emotions and illness to children had higher odds of initiating breastfeeding early [odds ratio (OR) = 2.42, 95% confidence interval (CI) = 1.30, 4.57] (Wren et al., 2015).

Studies between 1981 and 2016 consistently linked early termination of breastfeeding to mothers' fears that their milk was insufficient and that children should be trained to accept solid food (Brown et al., 2016; Cohen et al., 1999; Cohen, Haddix, Hurtado, & Dewey, 1995; Nieves et al., 1994; Olney et al., 2012; World Health Organization, 1981). Mothers stopped breastfeeding because of: beliefs around illness (Olney et al., 2012; Vemury & CARE, 1981), subsequent pregnancies (Brown et al., 2016) and use of contraceptives and medication (O'gara & Kendall, 1985).

Mother's beliefs about food determined the timing and types of foods that they provided. Beliefs around the ‘heating’ and ‘cooling’ properties of foods were reported in both older and newer studies. For example, giving oil or sugar water was believed to ‘cool the infant from the birth process’ (Izurieta & Larson‐Brown, 1995, p. 255) and prepare the child for breast milk (Vemury & CARE, 1981). Liquids such as herbal infusions were used before 24 months to ‘heat’ the child and protect against illness (Vossenaar, Garcia, Solomons, et al., 2012). Foods considered cooling (chicken, avocado, fruits, potatoes, beans, eggs and fatty foods) were avoided because they were believed to cause or exacerbate illness (Kincaid et al., 2013; Vossenaar, Garcia, Doak, & Solomons, 2012). During illness, some mothers felt that thick foods helped ‘plug up the child and stop the diarrhea’; others preferred thin foods ‘to replace liquid that is lost’ (Parker et al., 1998, pp. 9–10). A belief that malnourished children should be fed less frequently to avoid sickness was also reported (Izurieta & Larson‐Brown, 1995). In recent studies, some mothers linked supplements with increased child health; others perceived adverse effects such as diarrhoea (Garcia‐Meza, Gonzalez, Tumilowicz, & Solomons, 2017; Gonzalez et al., 2017; Olney et al., 2012).

Beliefs about intrahousehold food distribution, with a pro‐male bias, were discussed in two studies in 1979 and 2015. In one study, some mothers felt that high‐status items should be reserved for economic earners and greater quantities of these foods were given to boys rather than girls (Pigott & Kolasa, 1979). In another, perceptions that boys were hungrier than girls led to more frequent meals and earlier complementary feeding in boys (Tumilowicz et al., 2015).

Few studies investigated automatic motivations (*n* = 6), all published in the last decade. Studies mostly described children's tastes for food transfers or supplements. According to some mothers, children's dislike for meat or fortified cereal transfers reduced consumption of those foods (Newman et al., 2014). In one study, consumption of lipid‐based nutrient supplements did not differ by flavours provided (Matias et al., 2011); elsewhere, flavour was reported to limit use (Garcia‐Meza, Gonzalez, et al., 2017; Gonzalez et al., 2017; Olney et al., 2012).

#### 3.4.2 Social and physical opportunities

Social relationships were reported to influence child feeding in 16 studies between 1979 and 2017. Whilst husbands, relatives, other mothers, neighbours and health care workers were all identified as influencers, paternal grandmothers were consistently the most frequently discussed. Grandmothers were reported to encourage early introduction of other (non‐breast milk) fluids into child diets and dominated feeding decisions (Brown et al., 2016; Cohen et al., 1999; Garcia et al., 2012; Olney et al., 2012; Vossenaar, Garcia, Solomons, et al., 2012).

Lack of physical opportunities was highlighted in 17 studies published between 1979 and 2017, focusing mainly on poverty, women's lack of control over household finances, household access to foods and women's time. Recent studies identified poverty as a cause of delayed introduction of complementary foods beyond recommendations and low quantity and quality of complementary foods (Brown et al., 2016; Chary et al., 2011; Garcia‐Meza, Montenegro‐Bethancourt, et al., 2017). However, less income was not always associated with poorer feeding behaviours; one study reported that poverty increased colostrum feeding because women could not afford alternative foods for the child (Atyeo et al., 2017).

Access to foods, based on cost, accessibility and seasonality, influenced which foods were fed to children in three studies (Olney et al., 2012; Pigott & Kolasa, 1979; Vemury & CARE, 1981). Studies consistently found that mothers' work within or outside the home restricted their time for breastfeeding (Lutter, Pérez‐Escamilla, Segall, & Sanghvi, 1994; McKerracher et al., 2016) and their employment decreased exclusiveness and duration of breastfeeding (Lutter, Pérez‐Escamilla, Segall, & Sanghvi, 1994; McKerracher et al., 2016).

#### 3.4.3 Psychological and physical capabilities

Limited breastfeeding knowledge was a commonly cited constraint in studies across time points (between 1985 and 2017). Many women were unaware of the existence of colostrum (O'gara & Kendall, 1985), breastfeeding recommendations and techniques for ensuring sufficient milk supply (Cerezo & Claros, 1993; Gutiérrez Cabrera & Turcios España, 2004). One study found that mothers who were given information about breastfeeding by a health care professional were more likely to initiate breastfeeding early, but other studies found no association between knowledge sharing and breastfeeding practices (Atyeo et al., 2017; Dearden, Altaye, de Maza, de Oliva, Stone‐Jimenez, Morrow, et al., 2002).

Knowledge of healthy complementary feeding practices was also low, with confusion about how to use micronutrient supplements and a lack of awareness that malnourished children required more food (Engle & Nieves, 1992; Garcia‐Meza, Gonzalez, et al., 2017). Although one study reported good levels of knowledge, physical constraints and certain beliefs still prevented optimal practices (Olney et al., 2012).

Prenatal stress (higher vs. lower cortisol levels) was associated with delayed onset of lactation (3.0 vs. 2.4 days, *p* = 0.02) (Grajeda & Perez‐Escamilla, 2002). In some Guatemalan villages, mothers with delayed onset of lactation had increased risk of ending breastfeeding before 6 months (adjusted hazard ratio: 3.43, 95% CI = 1.55 to 7.59, *p* < 0.01), but the same association was not found in other villages (Hruschka et al., 2003) or in another study from Honduras (Perez‐Escamilla et al., 1996).

Six studies, published between 1977 and 2017, described child illness as a barrier to optimal feeding. Illness was associated with lower energy and protein intakes (Martorell et al., 1980; Mata et al., 1977) and lower intakes of nutritional supplements (Matias et al., 2011; Olney et al., 2012).

### 3.5 Evidence on IYCF interventions

We found 22 intervention studies reporting on 46 exposures, addressing all intervention categories in the Behaviour Change Wheel apart from coercion.

Most (*n* = 10) employed a combination of enablement and education techniques including food transfers, or provision of high‐energy or fortified supplements with behaviour change communication or nutrition counselling.

Studies from the 1990s aimed to determine optimal exclusive breastfeeding duration (Cohen et al., 1994), including for low‐birthweight children (Dewey et al., 1999), and to understand the effect of exclusive breastfeeding on food acceptance after 6 months (Cohen, Rivera, et al., 1995). Promotion of exclusive breastfeeding for the first 4 months, combined with provision of complementary foods after this (compared with encouraging exclusive breastfeeding for 6 months), was associated with the same or lower breastfeeding frequencies and decreased duration of breastfeeding and breast milk intake at 26 weeks (Cohen et al., 1994; Dewey et al., 1999). Exclusive breastfeeding to 6 months did not affect food acceptance at 9 or 12 months (Cohen et al., 1995).

More recent interventions used food assistance and education to encourage exclusive breastfeeding to 6 months and improve complementary feeding, with mixed success. A large‐scale food assistance and behaviour change communication programme [PROCOMIDA (Programa Comunitario Materno Infantil de Diversificación Alimentaria)] had minimal improvements among beneficiaries in exclusive breastfeeding or dietary diversity at midterm, despite improving knowledge (HDCi, 2013), but a cross‐sectional process evaluation showed improvements in early initiation of breastfeeding (94% vs. 74%) and dietary diversity (51% vs. 38%), compared with controls in 2012 (Olney et al., 2013). The final evaluation, based on longitudinal data between 2011 and 2015, showed smaller improvements in early initiation of breastfeeding (5 pp higher), increased exclusive breastfeeding at 6 months (11 pp) and some (mixed) improvements to complementary feeding (Heckert et al., 2018). Other combined interventions improved child diets: nutrition education provided with Plumpy'doz (lipid‐based nutrient supplement) increased energy intake (Flax et al., 2015) and education with fortified cereal product versus meat increased dietary diversity and the number of main meals but decreased the number of additional meals (Krebs et al., 2012).

Interventions using enablement techniques alone (five studies) primarily did so by providing food transfers or high‐energy nutrient supplements. Given to mothers, high‐energy (vs. low‐energy) supplements increased exclusive breastfeeding at 20 weeks postpartum (96% vs. 84%, *p* < 0.04) (Gonzalez‐Cossio et al., 1998). When given to children, provision of beans and corn (Martorell et al., 1979), a high‐protein energy drink (Islam & Hoddinott, 2009) and high‐energy cookies (Valverde et al., 1979) each increased energy intake.

Education alone, and combinations of education, training and social support interventions, had mixed effects on breastfeeding. More recent studies were not consistently more effective. Mother‐to‐mother support groups found no effects on early initiation or exclusive breastfeeding (Dearden, Altaye, de Maza, de Oliva, Stone‐Jimenez, Burkhalter, et al., 2002), but introducing maternity services increased exclusive breastfeeding (43% vs. 22% in comparison group) (Horton et al., 1996). ‘Baby‐Friendly Hospital’ activities, which include demonstrations of milk expression, lengthened exclusive breastfeeding in one institution but not in others (Lutter, Pérez‐Escamilla, Segall, Sanghvi, Teruya, & Rivera, 1994). Training health care professionals and introducing an information management system increased the percentage of children breastfed within the first 30 min (from 57% to 70%) (Pérez‐Escamilla, 2004). Of those targeting child diets, general nutrition education delivered to mothers was reported to ‘moderately’ improve children's vegetable and fruit intake (no statistics provided) (Asensio, 2013). However, individualised complementary feeding education, including one‐to‐one visits from community health workers and mother‐led goal setting, improved the percentage of children reaching minimum dietary diversity by 22% and minimal acceptable diet by 23% (Martinez et al., 2018).

### 3.6 Evidence on IYCF policies

We found evidence from seven policy studies, relating to communications/marketing, guidelines, regulation and service provision.

A national programme in Honduras, Project for the Support of Breastfeeding (PROALMA), aimed to improve breastfeeding practices by educating health care professionals and changing hospital policies, such as eliminating infant formula (Popkin et al., 1991). Evaluations found positive effects on prevalence of breastfeeding at 6 months (15 pp higher between programme and control) (American Public Health Association, 1987) and 12 months (35 pp increase between years 1982 and 1985) (Canahuati, 1990). Although initiation of breastfeeding was expected to decline due to secular changes, an increase of 2 pp was observed between 1981 and 1984, possibly as a result of PROALMA (Popkin et al., 1991). Another national programme, *Atención Integral a la Niñez en la Comunidad* (AIN‐C), led by the Honduran Ministry of Health, delivered growth monitoring and promotion activities, including tailored nutrition counselling. Midterm evaluation showed large effects on child feeding, particularly exclusive breastfeeding until 4 and 6 months (<4 months: 56% vs. 24%; <6 months: 46% vs. 19%) (Van Roekel et al., 2002). Subsequent evaluations showed sustained but smaller effects on breastfeeding practices (Schaetzel et al., 2008; Sierra et al., 2019).

As part of the Guatemalan government's Zero Hunger Pact Plan, a communication strategy was launched using sociodramas, community nutrition lotteries, television and radio programmes, to reduce child undernutrition (Grajeda & Campos, 2016). A qualitative evaluation suggested that child feeding knowledge improved more than practices (Grajeda & Campos, 2016).

### 3.7 Country‐level analysis

Overall, studies from Guatemala were more recently conducted, greater in number and of higher quality than those from Honduras and El Salvador. Themes we identified as sources of behaviour did not differ substantially by country or publication date. We did not conduct further analysis of between‐country differences due to the paucity of information on the same exposure–outcome pairings.

## 4 DISCUSSION

Our review maps evidence on the drivers of child feeding behaviours in the Northern Triangle and the effectiveness of related interventions and polices. We find a predominance of studies on mothers' beliefs and perceptions about food and breast milk, the influence of relatives (especially paternal grandmothers) and mothers' lack of resources on breastfeeding and child feeding practices. Interventions mostly provided food rations and high‐energy supplements nutritional education. We found only three evaluated policies. Evidence mapped onto the Behaviour Change Wheel reveals that, although there are pockets of well‐explored influences on child feeding, evidence is lacking on automatic reflections and capabilities. Evidence from El Salvador and in urban areas is also scarce. Intervention and policy evaluations were lacking in number, were not linked to the behavioural theory and many were not robustly evaluated.

### 4.1 Drivers of child feeding behaviours

The number of studies across the COM‐B categories appears to reflect the state of evidence in other countries, with comparatively more studies on reflective motivations and opportunities and fewer studies on automatic motivations and physical capabilities. This trend may mirror the relative importance of these categories in driving behaviours or may represent research gaps.

Themes within categories are also common to studies from other countries. For example, perceived milk insufficiency, lack of decision‐making power and gender bias have been reported to influence feeding practices in other countries (Basu, Aundhakar, & Galgali, 2014; Burns et al., 2016). Similarly, the importance of child willingness to be breastfed, illness and lactation problems has been highlighted elsewhere (Balogun, Dagvadorj, Anigo, Ota, & Sasaki, 2015).

These barriers reveal an important particularity of child feeding behaviours: to feed optimally, two sets of behavioural drivers must converge so that mother and child are willing and able to partake in child feeding (Ventura & Worobey, 2013). Furthermore, the motivations and capabilities of mothers and children may interact. For example, breastfeeding can strengthen the bond between mother and child, in turn acting as a predictor of continued feeding and milk supply (Agunbiade & Ogunleye, 2012).

Associations between psychological capabilities and child feeding are underresearched. Stress may be an important factor (Grajeda & Perez‐Escamilla, 2002), particularly in the Northern Triangle—a context among the most dangerous in the world (Runde, Perkins, & Nealer, 2016), with high occurrences of sexual violence (Latin America Working Group, 2016) and possibly maternal depression (Verbeek, Arjadi, Vendrik, Burger, & Berger, 2015). Numerous studies link stress and depression to poor child feeding outcomes (Fallon, Groves, Halford, Bennett, & Harrold, 2016; Madlala & Kassier, 2018), and some have linked intimate partner violence with child stunting (Rico, Fenn, Abramsky, & Watts, 2011) perhaps due to effects on child feeding.

### 4.2 Intervention and policies

Dietary behaviour change interventions in the Northern Triangle have typically justified their methods by citing evidence for their effectiveness in other settings, but rarely consider contextual or theoretical reasons for *why* the desired behaviour is not being carried out or *how* it is hypothesised to improve. These are common criticisms of behaviour change interventions and are important because they can limit intervention success (Aboud & Singla, 2012). Exceptionally, the formative research conducted for PROCOMIDA (Olney et al., 2012) identified the motivations of mothers, fathers and grandmothers, which may have contributed to its success.

Interventions providing resources may be more likely to improve child diets than breastfeeding. We found that poverty and food insecurity had more negative effects on child diets than breastfeeding, and the interventions in our review that gave mothers food or supplements tended to improve dietary diversity more than breastfeeding. Consistent with this, international reviews also find that food or cash transfers improve dietary diversity (Bastagli et al., 2016; Hidrobo, Hoddinott, Peterman, Margolies, & Moreira, 2014), but effects on breastfeeding indicators are more mixed (Bassani et al., 2013). This may be because the link between resources and breastfeeding is more complex, as poverty is associated with prolonged feeding (Chary et al., 2011) and the costs of breastfeeding are borne out more in terms of mothers' time and energy than the household food budget.

Particularly for breastfeeding, but also for complementary feeding, the provision of resources may be ineffective or insufficient (Bassani et al., 2013). Our review found that mothers' nutrition knowledge and beliefs guide child feeding behaviours, indicating that education and training are relevant techniques. Although education and counselling interventions in our review had mixed effects, international reviews find that they are highly effective at improving breastfeeding practices (Sinha et al., 2015) and have (smaller) effects on child diets (Webb Girard & Olude, 2012). Breastfeeding counselling interventions in the Northern Triangle might be made more effective by addressing the capabilities, opportunities and motivations identified in our review: for example, by discussing breastfeeding recommendations; the benefits of colostrum and breast‐milk; how mothers can know their milk supply is sufficient; feeding during illness and when taking medicines; and ways to continue feeding whilst working. Counselling on complementary feeding could discuss beliefs about heating and cooling foods, highlight benefits of diverse diets and supplements and explain how much to feed the child in general and during illness.

Given that cultural beliefs and influential figures were featured so frequently in our review, it is surprising that few interventions addressed these factors. Counselling interventions could aim to change social norms and opportunities by engaging relatives, especially paternal grandparents. Recent complex interventions that included actions designed to change social norms and engage key influencers have shown some success in other countries. In Ethiopia, the Alive and Thrive programme, which included counselling, with mass media and social mobilisation to engage other family members, improved exclusive breastfeeding by 9 pp (Kim et al., 2016). In India, Participatory Learning and Action community groups and home counselling, which engage the wider community, improved child minimum dietary diversity (OR = 1.47) (Nair et al., 2017). Interventions to address these social barriers could be tested alongside techniques to increase resources, knowledge and motivations.

A systematic search of policies addressing malnutrition across Latin America showed that all three Northern Triangle countries have implemented WHO‐recommended policies, including growth monitoring and promotion, regulation of the marketing of breast milk substitutes (part of the Baby‐Friendly Hospital Initiative) and paid maternity leave. Apart from the AIN‐C evaluations, our review demonstrates a lack of evidence on the impacts of these policies despite evidence that they have improved breastfeeding rates in other countries (Mirkovic, Perrine, & Scanlon, 2016; Sinha et al., 2015). None of the Northern Triangle countries have specific policies to address micronutrient deficiencies and there is insufficient resourcing of existing nutrition policies (Tirado et al., 2016), which may also explain the shortage of evaluations found in our review. The lack of monitoring and evaluation has been described as a barrier to progress in the region (Martorell, 2012) and must be a priority moving forwards.

### 4.3 Strengths and limitations

Our review benefits from a systematic search and duplicate assessment of study inclusion, quality and categorisation of studies onto the Behaviour Change Wheel. By focusing our review on the Northern Triangle, we restrict the generalisability of our findings, but this approach has enabled an in‐depth, context‐specific analysis of child feeding behaviours and demonstrates a mapping approach that could be applied in other contexts.

By using the COM‐B model, we found that categories overlapped and were difficult to disentangle—a challenge that would arise from applying any framework to a complex reality. For example, we found evidence that colostrum is withheld from children because of mothers' beliefs that it may cause illness. This belief could be categorised as mothers' reflections (reflective motivations), cultural norms (social opportunities) and/or a lack of knowledge (psychological capabilities). No studies related their findings to the COM‐B model, so our subjective categorisation of evidence is a potential source of bias. To ensure that evidence was mapped consistently, two reviewers independently categorised them, and we assigned multiple categories where necessary.

The choice of the Behaviour Change Wheel over another framework is another potential source of bias. The Behaviour Change Wheel places less emphasis on the stages of behavioural change than other frameworks, such as the transtheoretical model (Prochaska & Di Clemente, 1982). However, no studies in our review described these cognitive stages. Other nutrition‐specific frameworks unpack the direct, proximate and distal determinants of undernutrition (Black et al., 2008). However, they do not apply the behavioural theory or enable us to unpack the processes by which these determinants affect individual behaviours. We have confidence in our selection of the COM‐B framework because it is based on a systematic review of the behaviour change theory and is therefore very broad (Michie et al., 2011).

Because we were interested in understanding the capabilities, opportunities and motivations that explain behaviours, we excluded evidence on more distal determinants, which would require further assumptions to be made about the specific pathways to influencing individual behaviours. For example, differences in socio‐economic status may reflect differences in employment and therefore a mother's time to feed or financial resources to buy appropriate foods. Although distal factors can direct research to population groups, they do not explain the root causes of behaviour.

Given the range of study dates, designs and quality, it is difficult to determine to what extent our results reflect the current drivers of feeding behaviours and their relative importance across population subgroups. However, we do find consistency in results over time, and we highlight evidence gaps where we find them.

Finally, publication bias and reporting bias are possible limitations and are difficult to assess quantitatively due to the lack of studies on each exposure–outcome pairing.

To comprehensively understand the capabilities, opportunities and motivations of mothers and children to change feeding behaviours, we need recent high‐quality, mixed‐methods research in varied contexts within the Northern Triangle. This, along with evidence reviewed here, should be used with the behavioural theory to inform future interventions. These interventions require robust impact and process evaluations so effective approaches can be scaled up.

## CONFLICTS OF INTEREST

The authors declare that they have no conflicts of interest.

## CONTRIBUTIONS

MD and HHF developed the protocol. MD ran the searches, screened the results and assessed risk of bias. HHF duplicated screening and assessed risk of bias on a subsample of results. MD and HHF interpreted the evidence. MD wrote the first draft of the manuscript, with inputs from HHF.

## Supporting information

Table S1. Risk of bias assessment in quantitative studies of the influences on child feeding behaviours in Guatemala, Honduras and El Salvador (*n* = 38) using ROBINS‐I tool
Table S2. Quality assessment of qualitative studies of the influences on child feeding in Guatemala, Honduras and El Salvador (*n* = 16), using the CASP checklist
Table S3. Key findings of studies of the sources of child feeding behaviours in the Northern Triangle
Table S4. Key findings of studies of interventions and policies that aim to influence child feeding behaviours in the Northern Triangle
