# Supplementary material for: What influences child feeding in the Northern Triangle? A mixed‐methods systematic review
Source: Matern Child Nutr. 2020 May 26;16(4):e13018. doi: 10.1111/mcn.13018 (PMC7507456; doi:10.1111/mcn.13018)
Supplement: Supplementary file 1 — Table S1. Risk of bias assessment in quantitative studies of the influences on child feeding behaviours in Guatemala, Honduras and El Salvador (n = 38) using ROBINS‐I tool Table S2. Quality assessment of qualitative studies of the influences on child feeding in Guatemala, Honduras and El Salvador (n = 16), using the CASP checklist Table S3. Key findings of studies of the sources of child feeding behaviours in the Northern Triangle Table S4. Key findings of studies of interventions and policies that aim to influence child feeding behaviours in the Northern Triangle [file MCN-16-e13018-s001.docx]

Supplemental Table 1. Risk of bias assessment in quantitative studies of the influences on child feeding behaviours in Guatemala, Honduras and El Salvador (*n*=38) using ROBINS-I tool

| **Risk of Bias Domain** | **Not applicable** | **Low** | **Moderate** | **Serious** | **Critical** | **No information** |
| --- | --- | --- | --- | --- | --- | --- |
|  | *n* | *n* | *n* | *n* | *n* | *n* |
| Bias due to confounding | 0 | 12 | 1 | 5 | 20 | 0 |
| Bias in selection of participants | 0 | 23 | 3 | 4 | 3 | 5 |
| Bias in classification of interventions | 0 | 26 | 4 | 8 | 0 | 0 |
| Bias due to deviations from intended interventions | 12 | 12 | 2 | 7 | 0 | 5 |
| Bias due to missing data | 0 | 14 | 4 | 3 | 6 | 11 |
| Bias in measurement of outcomes | 0 | 6 | 25 | 5 | 0 | 2 |
| Bias in selection of reported results | 0 | 37 | 0 | 0 | 0 | 1 |
| **Overall risk of bias judgment** | **0** | **4** | **8** | **5** | **21** | **0** |

ROBINS-I tool (Risk of Bias in Non-Randomised Studies - of Interventions)

Supplemental Table 2. Quality assessment of qualitative studies of the influences on child feeding in Guatemala, Honduras and El Salvador (*n*=16), using the CASP checklist

| **Quality indicator** | **No** | **Unable to determine** | **Yes** | |
| --- | --- | --- | --- | --- |
|  | *n* | *n* | *n* | (%) |
| Was there a clear statement of the aims of the research? | 0 | 0 | 16 | (100) |
| Is a qualitative methodology appropriate? | 0 | 0 | 16 | (100) |
| Was the research design justified as appropriate to address the aims of the research? | 1 | 5 | 10 | (63) |
| Was the recruitment strategy justified as being appropriate to the aims of the research? | 3 | 7 | 6 | (38) |
| Were the data collected in a way that addressed the research issue? | 0 | 6 | 10 | (63) |
| Has the relationship between the researcher and the participants been adequately considered? | 1 | 10 | 5 | (31) |
| Have ethical issues been considered (informed consent and ethical approval)? | 1 | 8 | 7 | (44) |
| Was the data analysis sufficiently rigorous? | 0 | 7 | 9 | (56) |
| Is there a clear statement of findings? | 0 | 6 | 10 | (63) |

CASP= Critical Appraisal Skills Programme

Supplemental Table 3. Key findings of studies of the sources of child feeding behaviours in the Northern Triangle

| **Author (year)** | **Key findings of effects on IYCF behaviours** | **Assigned Behaviour Change Wheel categories** |
| --- | --- | --- |
| **Guatemala (*n*=30)** | |  |
| Atyeo et al., 2017 | **Giving colostrum:**   - Determined by belief that colostrum cleans the stomach, advice from healthcare workers, and lack of other foods to feed the infant*   **Withholding colostrum:**   - Colour of colostrum makes women fear it is dirty or makes children ill, midwives and mothers advising against giving 'yellow' milk   **EIBF:**   - Mothers given advice about breastfeeding from a healthcare professional, or another mother or relative were more likely to commence EIBF than mothers having no information (90% (healthcare professional) vs 71% (another mother or relative) vs 70% (no information), Chi-squared=6.01, *p*=0.05) | **Psychological Capability**  **Social Opportunity**  **Physical Opportunity**  **Reflective Motivation** |
| Brown et al., 2016 | **Child feeding (G):**   - Mother's perception that children remain hungry after breastfeeding increases the tendency to introduce complementary foods regardless of age - Subsequent pregnancies leads to weaning at any age due to mother's belief that breastfeeding while pregnant makes the child ill, “*once I get pregnant, I don’t give the child his milk anymore... I prefer to give atol to avoid getting him sick*” - Financial insecurity decreases diversity of child diets - Male control of household finances and beliefs of paternal grandmother dictated mothers’ food purchasing decisions | **Social Opportunity**  **Physical Opportunity**  **Reflective Motivation** |
| Chary et al., 2011 | **Child feeding (G):**   - Poverty induced by unstable job markets, employer corruption, husband's alcoholism, and husband withholding finances from the mother, causes insufficient quantity and quality of complementary foods | **Social Opportunity**  **Physical Opportunity** |
| Dearden et al., 2002 | **EIBF and EBF:**   - Receiving advice was not associated with EIBF. Receiving advice vs not: 15.3% vs 17.5% EIBF in control areas; 29.5% vs 23.7% in program areas - Receiving advice was not associated with EBF. Receiving advice vs not: 20.0% vs 24.2% EBF in control areas; 17.1% vs 19.5% in program areas - Giving advice also not associated with EIBF and EBF - Mother working outside of the home was significantly associated with decreased EBF: Working outside of the home vs not: 8.1% vs 20.7% EBF in control areas; 8.8% vs 24.5% in programme areas. - Of mothers who worked outside of the home, those who took the child to work continued EBF in 21.7% of cases in control areas and 15.4% in programme areas vs 0% and 3.2% respectively amongst mothers who did not take the child to work | **Psychological Capability**  **Social Opportunity**  **Physical Opportunity** |
| Engle and Nieves, 1992 | **Child feeding (G)**   - Feeding malnourished child with donated foods because of “*custom*” and “*the child likes the food*". Only two mothers mentioned low weight of the child and only 5% of mothers specified the foods as “*special to give to the child*” or that the “*food was for the malnutrition of the child*" - No mothers spontaneously discussed giving more food to the malnourished child or that distribution was influenced by gender - Malnourished children of mothers who expressed preferences for equal household food allocation received a higher proportion (statistics not reported) of the families protein (t=2.19, *p*<0.03) compared to other expressed preferences | **Psychological Capability**  **Reflective Motivation** |
| Garcia et al., 2012 | **Child feeding (G)**   - Intergenerational beliefs are passed to mothers from grandmothers, mothers and aunts, including beliefs about certain foods (specific foods not reported) and their ability to protect children from illness and suffering, and a,belief that the child will innately prompt the mother to give certain foods (specific foods not reported)* | **Social Opportunity**  **Reflective Motivation** |
| Garcia-Meza et al., 2017 | **Feeding micronutrient powders and supplements:**   - Facilitated by mothers’ perception of increased health and appetite of the child - Inhibited by confusion over instructions for the supplement, perceived adverse secondary effects for child (specific effects not reported), and the flavour | **Psychological Capability**  **Automatic Motivation**  **Reflective Motivation** |
| Garcia-Meza et al., 2017 | **Delayed complementary feeding:**   - Perceived fussiness of the child - Fear of causing diarrhoea, indigestion or other negative effects - Negative experiences and advice of role models including older siblings and grandmothers - Financial insecurity | **Social Opportunity**  **Physical Opportunity**  **Reflective Motivation** |
| Gonzalez et al., 2017 | **Use of home-fortification product (SQ-LNS):**   - Barriers to consuming SQ-LNS for children include a dislike of the flavour (peanut), rejection and large portion size - Barriers to feeding SQ-LNS for caregivers include difficulty in finding the right food to mix with, lack of patience to feed, activities outside the house, father not collaborating, and perceived adverse effects e.g. diarrhoea and constipation - Perceived benefits included the prevention of illness, and increased appetite, activity, wellbeing and weight gain | **Social Opportunity**  **Physical Opportunity**  **Automatic Motivation**  **Reflective Motivation** |
| Grajeda and Pérez-Escamilla, 2002 | **Onset of lactation:**   - Within the primiparous group, salivary cortisol levels were not associated with onset of lactation but amongst multiparous women, a salivary cortisol level >16.55 nmol/L experienced onset of lactation later that their counterparts (Adjusted mean onset of lactation=3.0 days postpartum, SE=0.2 days vs mean=2.4 days postpartum, SE= 0.2 days respectively) - No evidence of an association between cervical dilation and onset of lactation (coefficient=0.025, SE=0.037, *p*=0.495) | **Physical Capability**  **Psychological Capability** |
| Hruschka et al., 2003 | **Ending full breastfeeding (either EBF or predominant breastfeeding):**   - In two villages delayed onset of lactation (>3 days vs ≤3 days) was associated with ending breastfeeding <6m, but no evidence for an association was found in other villages.   - Village A: Adjusted hazards ratio (AHR) =2.87 (95% CI 1.25, 6.60), *p*<0.05   - Village B: AHR=3.43 (1.55, 7.59) *p*<0.01;   - Village C: AHR=0.26 (0.06, 1.14) *p*>0.05;   - Village D: AHR=1.11 (0.44, 2.77) *p*>0.05 - Women who did not experience onset of lactation by day 2 postpartum, compared to those that did, were more likely to begin supplementation with formula or cereal-based fluids on day 3 (as opposed to later) (OR=2.04, 95% CI=1.06, 3.92) and on day 4 (as opposed to later) (OR=3.85, 95% CI=1.21, 12.27) | **Physical Capability**  **Psychological Capability** |
| Immink and Alarcon, 1993 | **Energy and protein intake:**   - Households with higher per capita incomes (top tercile) were less likely to have energy deficient (Probit coefficient=-0.163, *t-*ratio=2.44, *p*<0.05) and protein deficient diets (Probit coefficient=-0.308, *t*-ratio=4.06, *p*<0.01) - Increasing monthly per capita expenditures on food was associated with a decreased risk of energy deficient diets in pre-schoolers (≤5 years) (Probit coefficient= -0.020 *t*-ratio=2.90, *p*<0.01) | **Physical Opportunity** |
| Izurieta and Larson-Brown, 1995 | **Introduction of supplementary foods and meal frequency:**   - During first 3 days after birth, children were reported to be given small amounts if oil or sugar water, because of cultural perceptions that it will “*clean the baby inside*” and to “*cool*” the child from the stress being born* - Mothers with malnourished children aged between 7-12m were reported to feed them only 1-2 times per day because of cultural beliefs that “*children are small so they need to eat less often*” and “*if they eat too much they can get sick*”* | **Social Opportunity**  **Reflective Motivation** |
| Kincaid et al., 2013 | **Child feeding (G):**   - Complying with infants’ demands for food was believed to protect against parasites, even within the first 6m* - Foods such as fruits, chicken, avocado and potatoes were culturally perceived to be linked with gastrointestinal illness in infants* | **Social Opportunity**  **Reflective Motivation** |
| Martorell et al., 1980 | **Energy and protein intake:**   - Illness was associated with a decrease in average energy and protein intake per day:   - Respiratory illness: Energy=-67kcal (*p*<0.001), Protein=-1.0g (*p*=0.007).   - Diarrhoea: Energy=-160kcal (*p*<0.001), Protein=-3.0g (*p*<0.001).   - Apathy: Energy=-175kcal (*p*<0.001), Protein=-5.1g (*p*<0.001) - Within-child comparisons showed an average decrease in energy when the child went from being healthy to sick by 148kcal/day and an increase when a child went from being sick to healthy by 125kcal/day (CI and p-value not given) | **Physical Capability** |
| Mata et al., 1977 | **Energy and protein intake:**   - Increased total illness was associated with decreased average of recommended calorie intake (coefficient=-0.4041, *p*=0.015) and the average of the child's recommended protein intake (coefficient=-0.3446, *p*=0.034) | **Physical Capability** |
| Matias et al., 2011 | **Consumption of lipid-based micronutrient supplement (LNS):**   - Consumption did not differ by LNS flavour (*p*=0.35) during a three day feeding trial - Similarly there was no difference by flavour in the two-week home-feeding trial 74.6 ± 20.0 vs 67.6 ± 29.2, (*p*=0.639) - Average feeding duration of LNS (minutes) did not differ by LNS flavour (*p*=1.00) - Percentage of LNS supplement consumed was negatively associated with percentage of days child reported to have no appetite (coefficient= −0.43, *p*<0.001), diarrhoea (coefficient= −0.22, *p*=0.042), and fever (coefficient= −0.28, *p*=0.008) | **Physical Capability**  **Automatic Motivation** |
| McKerracher et al., 2016 | **Duration of breastfeeding:**   - Duration of breastfeeding (m) was negatively associated with reported reliance on higher levels of help with child feeding and care (coefficient= -2.845 (95% CI -5.158, -0.531), *p*<0.01) and working away from the home (coefficient= -4.989 (-9.486, -0.312), *p*<0.05) - Breastfeeding duration was not associated with the reported preferred use of high-quality, protein-rich foods | **Social Opportunity**  **Physical Opportunity**  **Automatic Motivation**  **Reflective Motivation** |
| Newman et al., 2014 | **Consumption of meat and micronutrient fortified cereals:**   - Reasons given by caregivers for why the child did not consume the food provided in the intervention or comparison group were: illness, lack of time, and the child not liking the food - The proportions of days without the food accounted for by these reasons were slightly (but not significantly) higher in the meat group than the cereal group (38% vs 32%, 31% vs 14% and 12% vs 3% respectively) | **Physical Capability**  **Physical Opportunity**  **Automatic Motivation** |
| Olney et al., 2012 | **Breastfeeding:**   - Knowledge and beliefs were generally supportive of positive breastfeeding practices, including those of fathers and grandmothers. Barriers included beliefs of insufficiency of breast milk, use of other liquids to 'quench thirst' during illness and coffee as a treatment for diarrhoea.* Less common barriers were: the child not asking for food, subsequent pregnancies, and work outside the home   **Complementary feeding:**   - Knowledge was generally good. Barriers included cost and local availability, fears of causing child illness and beliefs of the father and grandmother, actual child illness, mothers' concerns over readiness to consume meat   **Use of lipid based supplements:**   - Facilitated by mothers' knowledge that LNS could improve their own health and that of the infant, and mothers' approval of the taste and smell. Barriers included a lack of foods to mix LNS with, lack of money, illness of mother or infant, beliefs of father, child dislike, mother's dislike or mistrust, perceived adverse effects including nausea, vomiting, wheezing and crying more, mothers time, and lack of knowledge on how to use supplements | **Capability (Physical and Psychological)**  **Motivation (Automatic and Reflective)**  **Opportunity (Social and Physical)** |
| Parker et al., 1998 | **Consistency of complementary foods:**   - Mothers preferred to give thicker foods to older children in order to "*fill the child*" and "*so that it sustains the stomach*" - No strong differences were shown when children were healthy but when they had a cough or fever, 78% of women preferred to give thin complementary foods. "*The complementary food should be very thin so that the child will eat it and so that it will go down the throat easily*" - When children had diarrhoea, particularly at 6m, a dichotomy of preferences emerged with 35% of mothers preferring thick foods and 48% preferring thin/liquid foods. Some felt thick foods were important "*because it helps to plug up the child and stop the diarrhoea*" and "*because it hardens the stomach*", also believing that thin foods may make the child worse. Others preferred thin/liquid foods "*to replace the liquid that is lost in diarrhoea*" and "*when they have diarrhoea, they are thirsty, and because of this they will take a little food if it is thin*." | **Physical Capability**  **Reflective Motivation** |
| Pigott and Kolasa, 1979 | **Child feeding (G):**   - The most frequently grown crops determined the staples of child diets: beans, maize, tortillas and coffee. Fruits were added when the family’s trees produced them. Fresh cheese, eggs, rice, sweet rolls, cabbage, potatoes and guiquil were dietary additions depending on financial resources - Meat, milk and canned goods were considered high status items and more commonly reserved for economically productive members of the family - typically men. These foods were also given in larger quantities to boys rather than girls* | **Social Opportunity**  **Physical Opportunity**  **Reflective Motivation** |
| Solien de González, 1964 | **Child feeding (G):**   - Cultural beliefs about 'hot', 'cold', 'strong' and 'indigestible' foods considered appropriate for certain personality types or physical constitutions was reported to prevent foods such as meat, potatoes, black beans, corn, and powdered milk being given to young children* | **Social Opportunity**  **Reflective Motivation** |
| Tumilowicz et al., 2015 | **Child feeding (G):**   - Mothers’ beliefs that boys are hungrier and less easily satisfied than girls was reported to cause mothers to feed boys more frequently and introduce supplementary snacks. “*Well, here, the boy always eats more. You have to give [the boy] a snack. Sometimes, when you breastfeed him, well, he always stays fussy, and if you give him a snack, he stays satisfied, sometimes boys eat up to 4 times per day! In comparison, the girl eats 3 times! The girl eats less than the boy.*”* | **Social Opportunity**  **Reflective Motivation** |
| Vemury & CARE, 1981 | **Child feeding (G):**   - Mothers’ beliefs that honey and oil given to newborns is cleansing (14.0% of mothers reported this), custom (10.8%), ‘strength giving’ (6.8%), and prepares the baby to receive milk (3.3%)* - 56.3% of mothers reported that they do not breastfeed when ill and 13.5% reported that they breastfeed less than usual. 20% of mothers were reported to withhold the breast completely when the child is ill, predominantly because they believed that the child needs rest or that the milk aggravates the illness - Reported reasons for early introduction of complementary foods included: new pregnancies, the belief that the child needs more food, and perceptions that they had insufficient milk - Foods believed to be beneficial included: boiled milk (believed by 78% of mothers), eggs (37%), Incaparina (a high-protein supplementary food) (42%) and dry beans were considered harmful by 20%. Most mothers fed their children according to these beliefs, though some cited cost and availability as major constraints* | **Social Opportunity**  **Physical Opportunity**  **Reflective Motivation** |
| Vossenaar et al., 2012 | **Child feeding (G):**   - During illness, increased amounts of liquid were offered to children 0-23m, in the form of water, ritual fluids (such as herbal infusions or broths), breast milk, or oral rehydration solutions were offered by 79% of mothers - Many mothers reported withholding foods such as beans, eggs, fatty-foods and other items during child illness. These foods are culturally believed to 'cool' the body and are not considered appropriate* - Mothers encouraged children to consume foods including soups, broths, ritual fluids, foods low in fat and breast milk during illness | **Social Opportunity**  **Reflective Motivation** |
| Vossenaar et al., 2012 | **Early introduction of *agüitas* (liquids given to infants):**   - Mothers-in-law encouraged the introduction of *agüitas,* and doctors were also reported to influence mothers' decisions - Different ritual fluids were reported to have various perceived benefits: to alleviate stomach pain, colic, and constipation, other health benefits (heating the infant, and treating sore throat, lungs, fever, worms)* | **Social Opportunity**  **Reflective Motivation** |
| Wehr et al., 2014 | **Child feeding (G):**   - Cultural beliefs reported regarding strict feeding times and the importance of not feeding the child too much. These beliefs were reported to be strongly influenced by other females, especially paternal grandmother* - Child feeding was reported to be affected by mothers’ lack of financial control, was reported to be often strongly influenced by paternal grandmothers | **Social Opportunity**  **Physical Opportunity**  **Reflective Motivation** |
| World Health Organization, 1981 | **Child feeding (G):**   - Within an economically advantaged group of mothers (EA) and an economically disadvantaged urban group (ED), the reasons for not breastfeeding included: child ill in hospital (EA=6%, ED=20%) or does not suckle (EA=6%, ED=10%), no or insufficient milk (EA=43%, ED=37%), breast and nipple problems (EA=9%, ED=3%), mother ill (EA=4%, C=2%), emotional problems or beliefs (EA=13%, ED=2%), does not want to (EA=13%, ED=3%) - Mothers reported reasons for regular dietary supplementation with complementary foods that included “*to improve growth*”, “*to feed better*”, *“to get the child used to it*”, “*child old enough*” and “*child wanted other foods*” and at 3m postpartum 10-17% of mother said that they thought their milk was insufficient | **Physical Opportunity**  **Reflective Motivation** |
| Wren et al., 2015 | **EIBF:**   - Children born to mothers who don't believe in *susto* (transmitting negative emotions and illnesses to the child through the breast milk), vs those that do, were more likely to receive EIBF (OR=2.42, (95% CI 1.30, 4.57), *p*=0.01) | **Social Opportunity**  **Reflective Motivation** |
| **Guatemala and El Salvador n=1** | |  |
| Nieves et al., 1994 | **Child feeding (G):**   - Reported by authors to be influenced by negative beliefs about colostrum in most Guatemalan women and about half of women from El Salvador - Mothers encouraged to introduce liquids other than breast-milk by people around her (e.g. healthcare workers or paternal grandmothers) - Child feeding reported to be influenced by mothers’ own beliefs that babies require other liquids to quench thirst, and that breast milk is insufficient, contaminated by emotional states or that physical exertion of the mother will result in her breast-milk becoming bad and the child becoming ill* - Mothers reported beliefs that children become sick and malnourished if not given extra foods. Economic and material constraints of working and feeding the rest of the family also reported to influence child feeding | **Social Opportunity**  **Physical Opportunity**  **Reflective Motivation** |
| **El Salvador n=1** | |  |
| Cerezo and Claros, 1993 | **Breastfeeding (G):**  Authors describe the following factors as barriers to optimal breastfeeding, identified through interviews with mothers:   - Lack of information given to mothers during pregnancy about child feeding by healthcare workers - Mothers were unaware of recommendations for EBF duration - Mothers had difficulty in understanding that if the child is unsettled or sleepless, this can be caused by other factors, not just the need for food - Near complete lack of knowledge about what mothers need to do to ensure sufficient milk production - Mistaken beliefs were reported that children require boiled water before 4m - Belief that breastfeeding makes the breasts ugly | **Social Opportunity**  **Psychological Capability**  **Reflective Motivation** |
| **Honduras n=7** | |  |
| Cohen et al., 1995 | **Interruption of EBF:**   - Reasons given: 45% of mothers believed the child 'needed' more food, 'asked' for food, or was ready, 18% to ‘acclimate' the infant's stomach, 6% to teach the child to differentiate flavours, 12% felt solid food sustains the child more than breast milk, 6% felt they were not producing enough milk, 3% returning to work | **Reflective Motivation**  **Physical Opportunity**  **Social Opportunity** |
| Cohen et al., 1999 | **Interruption of EBF:**   - Mothers perceived their breast-milk to be insufficient - Mothers perceived that the child required additional foods to breast-milk - Advice given to mothers from neighbours and relatives, their own mother, other family members, the husband and mother-in-law | **Social Opportunity**  **Reflective Motivation** |
| Gutiérrez Cabrera and Turcios España, 2004 | **Any breastfeeding:**   - Reasons given: 94% of women thought that breast-milk was nutritious - Knowledge of recommendations: 46/ 49 women who knew the minimum recommended duration of breastfeeding were breastfeeding; 118/ 128 women who were not aware of recommendations were breastfeeding (no statistical comparison) | **Psychological Capability** |
| O'Gara and Kendall, 1985 | **Breastfeeding and bottle-feeding:**   - 3% of women were aware that colostrum contains protein and antibodies and 40% of women were unaware of its existence. - Mothers describes colostrum as dirty, thin, thick, sticky, ruined, old, agitated, hot, stored, yellow, brown, bad - Belief that of breast milk may be contaminated by certain medications and contraceptives, emotions or work and the belief that anaemic mothers should not breastfeed* - Mothers reported social pressures to stop breastfeeding completely after 6-8m. - Bottle-feeding driven by desire to have a healthy (fat) baby, to be seen to be assuming responsibility by peers, partner jealousy, to enjoy city living and continue working, fear of becoming pregnant again | **Psychological Capability**  **Social Opportunity**  **Reflective Motivation** |
| Lutter et al., 1994 | **EBF and introduction of other milks and solids:**   - Current employment of mothers at 1 and 2m postpartum compared to mothers not employed was associated with a higher prevalence of breastfeeding with other milks (60.5% vs 33.4% *p*<0.001), breastfeeding with solids (7.9% vs 2.6% p=0.05), giving bottle (90.0% vs 56.0% *p*<0.001), and decreased prevalence of EBF (15.8% vs 39.5% *p*<0.01) | **Social Opportunity**  **Physical Opportunity**  **Reflective Motivation** |
| Pérez-Escamilla et al., 1995 | **EBF:**   - Mothers who planned to EBF for ≥3m had a probability of 0.44 of EBF at 1m and 0.35 at 2m. Mothers who planned to EBF for <3m had a probability of 0.25 of actual EBF at 1m and 0.17 at 2m. - Cox regression coefficient of planned EBF duration ≥3m vs <3m = 0.513, SE=0.084, *p*<0.001 | **Reflective Motivation** |
| Pérez-Escamilla et al., 1996 | **EBF and any breastfeeding:**   - Earlier milk arrival (day 3 postpartum or earlier vs day 4 postpartum or later) was positively associated with EBF (OR=1.41, 95% CI=0.55 to 3.64)) but not with any breastfeeding (OR=0.78, 95% CI=0.19 to 3.11) | **Physical Capability**  **Psychological Capability** |

Notes: AHR=adjusted hazards ratio; EA=economically advantaged; EBF=exclusive breastfeeding; AD=economically disadvantaged; EIBF=early initiation of breastfeeding (within 1 hour postpartum); (G)-general topic of discussion; m=months; LNS=lipid-based micronutrient supplement; OR=odds ratio; SE=standard error; SQ-LNS=small quantity lipid-based nutrient supplement; LNS= lipid-based micronutrient supplement. *Cultural beliefs have been coded as social opportunities and reflective motivations as they represent the beliefs of a mother and those around her.

Supplemental Table 4. Key findings of studies of interventions and policies that aim to influence child feeding behaviours in the Northern Triangle

| **Author (date)** | **Study design**  **and description of activities** | **Key Findings** |
| --- | --- | --- |
| **Interventions** | | |
| **Guatemala (*n*=14)** | | |
| Asensio, 2013 | Cluster control trial: Intervention participants in one township received anti-parasitic medication, multivitamins, educational messages, and home-prepared recipes. Another township acted as control | "Positive feeding behaviours and consumption of a diverse foods including fruits and vegetables was moderately improved" (No data reported) |
| Dearden et al., 2002 | Program impact evaluation using repeated cross-sectional surveys:  *La Leche League*'s Breastfeeding promotion program providing counsellor-facilitated mother-to-mother support groups | There was no difference in the change in the prevalence of women initiating breastfeeding within first hour between program and control groups (+1.0% and +3.0% respectively). Changes in EBF rates also did not differ between groups.  However, a dose-response effect was observed: mothers receiving both home visits and attending support groups breastfed in the program communities had higher prevalence of EBF (45%) than women in the program communities who did not receive these intervention components (14%). |
| González-Cossío et al., 1998 | Randomised controlled trial: Intervention was a high-energy supplement, controls received a placebo | Mothers given high-energy supplements were more likely to EBF at 20wks compared to mothers given a low-energy supplement (96% vs 84%, *p*<0.04) but by 25 weeks this effect only remained in undernourished mothers (*p*<0.05) |
| Health and Development Consulting International (HDCi) LLC, 2013 | PROCOMIDA (Programa Comunitario Materno Infantil de Diversificación Alimentaria) providing food assistance and behaviour change communication (BCC) on nutrition and health to reduce child undernutrition  All intervention groups received BCC and different arms received different family food rations: full family ration (FFR), reduced family ration (RFR) or no family ration (NFR) and different individual rations: corn-soy blend (CSB), lipid-based nutrient supplement (LNS), micronutrient powder (MNP), compared to a control group that received neither BCC nor food rations  Midterm compared with baseline, using repeated cross-sectional surveys | PROCOMIDA had little or no beneficial effect on child feeding at midterm evaluation  Indicators comparing midterm levels to baseline within PROCOMIDA beneficiary group: - Decreased EBF 0-6m (60.8% vs 65.1%, no statistical test performed). - Increased % children aged 6-24m with minimum acceptable dietary diversity (63.2% vs 60.1%, no statistical test performed) - There was no evidence for an association between increased nutrition knowledge amongst mothers and increased dietary diversity in children (*p*=0.948) or EBF (*p*=0.686) |
| Heckert et al. 2018 | PROCOMIDA (Programa Comunitario Materno Infantil de Diversificación Alimentaria) providing food assistance and behaviour change communication on nutrition and health to reduce child undernutrition  All intervention groups received BCC and different arms received different family food rations: full family ration (FFR), reduced family ration (RFR) or no family ration (NFR) and different individual rations: corn-soy blend (CSB), lipid-based nutrient supplement (LNS), micronutrient powder (MNP), compared to a control group that received neither BCC nor food rations  Impact evaluation using longitudinal cohort household surveys | PROCOMIDA interventions improved most breastfeeding outcomes but inconsistent effects were seen for other IYCF indicators  PROCOMIDA intervention arms showed significantly improved practices compared to controls for the following indicators (data taken from forest plot graphs so percentage points (pp) are estimates - significance based on plotted 95% confidence intervals):   - EIBF by 5pp in pooled intervention effect - EBF at 4m by 9pp, at 6m by 11pp in pooled effects - Predominant breastfeeding at 4m by 4pp, at 6m by 9pp in pooled effects - Minimum meal frequency by 5pp at 24m in pooled effects and for all intervention groups except NFR - Total number of food groups consumed in last 24hrs (around 0.2 food groups) in FFR+CSB at 9, 18 and 24m and in RFR+CSB at 24m (including CSB usage), in FFR+LNS at 18m and FFR+MNP at 18m and 24m - % children reaching minimum dietary diversity in past 24hrs in FFR+CSB (including CSB) at 12 and 24m by 7pp, FFR+LNS at 18m by 8pp and FFR+MNP at 12, 18 and 24m by 5-7pp. - Minimum acceptable diet in past 24hrs at 24m in FFR+CSB (including CSB usage) by about 7pp and in FFR+MNP by about 13pp   **No significant effects of PROCOMIDA, compared to controls, on:**   - EBF at 1m in all intervention groups, except FFR+MNP by about 4pp - Predominant breastfeeding at 1m, except RFR+CSB by about 3pp - % children breastfed in the past 24hrs, except FFR+MNP at 12m by about 1pp - % children who received semi solid foods in past 24hrs at 6 or 9m, although effect estimates were between about -6pp and -12pp for all intervention arms at 6m - Consumption of iron-rich foods, not including rations - % children receiving minimum acceptable diet in RFR and NFR - Minimum meal frequency at 6m, 9m, 12m and 18m for pooled effects and all intervention groups except FFR+LNS at 18m (4pp) and FFR+MNP at 12m (3pp) - Total number of food groups consumed at 6, 9 and 12m, not including the use of CSB - % children reaching minimum dietary diversity in all groups at 6 and 9m, and at all time points in RFR+CSB and NFR+CSB - Minimum acceptable diet at all time points in RFR+CSB, NFR+CSB, FFR+LNS, except 18m in FFR+LNS by about 7pp |
| Islam and Hoddinott., 2009 | Secondary data analysis of a cluster-randomised controlled trial: Intervention participants received Atole (a high-protein energy drink). Controls got a low-calorie drink. | On average children in intervention group consumed 101 kcal more per day as a supplement (*p*<0.01), 45 kcal less at home following supplement, and 56 kcal more in total per day (including supplement use and food consumed at home), compared with controls |
| Kennedy et al., 1994 | Comparative analysis of case studies: *Cuatro Pinos* farmers cooperative adopting export vegetable production | Average age at which child was weaned did not differ between groups (16.2m vs 16.2m, no statistical tests performed) |
| Krebs et al., 2012 | Cluster randomised trial: Provision of meat, compared with equi-caloric micronutrient-fortified rice-soy cereal product | Breastfeeding and bottle-feeding did not differ by treatment group at any time points (9, 12, 18m). Children in cereal groups consumed more main meals at 9, 12, 18m but fewer additional meals (*p*<0.05). Meat group consumed significantly more food groups at 9, 12m but not at 18m. |
| Martinez et al., 2018 | Randomised controlled trial: Delivered to mother-child dyads assigned to receive either standard care, consisting of generic age-based complementary feeding messages delivered by community health workers (control), or the intervention consisting of standard care plus individualised complementary feeding education delivered through structured interviews, 24-hour dietary recalls and open-ended goal-setting questions | In the intervention group, the following indicators were improved compared to control: - Minimum dietary diversity by 22% (*n*=135 (93.1%) vs *n*=115 (76.2%), RR=1.22, 95% CI=1.11 to 1.35)  - Minimal acceptable diet by 23% (*n*=123 (84.8%) vs *n*=104 (68.9%), RR=1.23, 95% CI=1.08 to 1.40)   But no improvement was seen for: - Minimum meal frequency (*n*=129 (89.0%) vs *n*=131 (86.8%) RR=1.02, 95% CI=0.94 to 1.12) |
| Martorell et al., 1979 | Before-and-after study:  Provision of food ration (100g beans and 90g corn per day per individual) | Energy intake (mean kcal/day ± SD) for children <6 years increased during the intervention (Before: 737 ±349, After:935±380, Change=198, *p*≤0.001). The change was significant for children aged 0-23m (change=235 *p*≤0.01) and for children aged 48-72m (change= 245, *p*≤0.001) but not those aged 24-47m.  Protein intake (mean g/day ± SD) for children under 6yrs increased during the intervention (Before: 20.9±10.6, After: 26.7±10.8, Change:5.8, p≤0.001). The change was significant for children aged 24-47m (change=4.4, *p*≤0.05) and for children aged 48-72m (change:7.8, *p*≤0.05) but not aged 0-23m. |
| Olney et al., 2013 | PROCOMIDA (Programa Comunitario Materno Infantil de Diversificación Alimentaria) providing food assistance and behaviour change communication on nutrition and health to reduce child undernutrition  All intervention groups received behaviour change communication and different arms received different family food rations: full family ration, reduced family ration or no family ration and different individual rations: corn-soy blend, lipid-based nutrient supplement, micronutrient powder, compared to a control group that received neither behaviour change communication nor food rations  Process evaluation using cross-sectional design with random sampling, mixed methods | PROCOMIDA had positive effects on breastfeeding and dietary diversity but did not reduce the number of participants delaying introduction of complementary foods beyond 6m during process evaluation  Positive effects of PROCOMIDA intervention were seen in the following indicators, compared to controls:   - 82% of beneficiary women perceived a positive impact of PROCOMIDA on child diets, related to having more food available and more diverse diets - Increased likelihood of children meeting minimum dietary diversity in past 24hrs (51% vs 38%). - Increased EIBF (60/64, 94% vs 14/19, 74%) - Reduced introduction of liquids other than breast milk before 6m (5/69, 7% vs 4/15, 27%).   No evidence for an effect of PROCOMIDA activities on:   - Likelihood of delaying introduction of complementary foods after 6m (13% vs 16%) - Feeding practices during illness |
| Olney et al. 2018 | PROCOMIDA (Programa Comunitario Materno Infantil de Diversificación Alimentaria) providing food assistance and behaviour change communication on nutrition and health to reduce child undernutrition  All intervention groups received BCC and different arms received different family food rations: full family ration (FFR), reduced family ration (RFR) or no family ration (NFR) and different individual rations: corn-soy blend (CSB), lipid-based nutrient supplement (LNS), micronutrient powder (MNP), compared to a control group that received neither BCC nor food rations  Impact evaluation using cluster-randomised controlled trial | Use of individual rations in CSB, LNS and MNP groups, varied by family ration size and type of individual ration  Size of family ration:   - Compared to groups with no family ration, in groups receiving full family ration more children had used CSB in the last 24hrs in all age groups (6m: 20.97% vs 14.63%, *p*<0.01; 9m: 40.28% vs 23.25%, *p*<0.01; 12m: 38.78% vs 22.51%, *p*<0.01; 18m: 43.75% vs 26.38%, *p*<0.01; 24m: 38.37% vs 17.53%, p<0.01) and had higher mean number of days of CSB usage in the last week (Mean±SD: 6m: 0.96±1.96 vs 0.54±1.46, p<0.05; 9m 2.03±2.52 vs 1.09±1.99, p<0.01; 12m: 1.99±2.41vs 1.11±2.0, p<0.01; 18m; 2.08±2.42 vs 1.17±2.10, p<0.01; 24m: 1.71±2.21 vs 0.88±1.83, p<0.01) - Compared to groups with reduced family ration size, in groups receiving full family ration more children had used CSB in past 24hrs, and had a higher mean number of days of CSB usage in the last week amongst all age groups but differences were not statistically significant (*p*>0.05).   Type of individual ration:   - At 9, 12, 18 and 24m more children in LNS and MNP groups had used their assigned individual ration in the past 24hrs (51–66%) than those in the CSB group (38–44%). And more frequently in the past week (Mean±SD: LNS and MNP: 3.13±3.08 to 4.35±3.11; CSB: 1.71±2.21 to 2.03±2.52). - Between LNS and MNP groups, children were more likely to have used their assigned ration in LNS at 9m (65.82% vs 51.11%, *p*<0.01) and more frequently at 9m (4.22±3.14 vs 3.13±3.08, *p*<0.01) and 12m (4.15±3.16 vs 3.50±3.07, *p*<0.05) but no differences were found at 18 or 24m. |
| Sosa et al., 1976 | Randomised controlled trial:  Intervention mothers were left alone with their newborn for 45mins and encouraged to breastfeed. Control participants were separated from their child until 12h postpartum | Varying associations between early skin-to-skin contact and duration of breastfeeding across sites, with 50 days (*p*<0.1) and 92 days (*p*<0.05) longer breastfed in experimental group for sites 2 and 3 respectively, but 100 days shorter in site 1 (*p*<0.01): % of women breastfeeding was also consistently higher in experimental group than controls in Site 2 and 3 |
| Valverde et al., 1979 | Before-and-after study: Participants were given two high-energy cookies daily for 4 weeks | Average increase in total energy intakes was 178 kcal (SD=225, *p*<0.01) in children, but no difference protein or food item intakes. |
| **Honduras (*n*=7)** | | |
| Cohen et al., 1994 | Three-arm randomised trial:  (1) EBF vs (2) introduction of complementary foods at 4m, with *ad lib* nursing 4-6m (SF), vs (3) introduction of complementary foods at 4m, with maintenance of baseline nursing frequency 4-6m (SF-M).  Mothers were provided with complementary foods. | Reductions in nursing frequency and duration between 16-26 weeks were greater in the SF and SF-M groups than in the EBF group.   - Changes in average times/day (SD) at 16-26w: EBF=0.2 (2.6), SF 2.4 (2.6) and SF-M 0.5 (2.0) fewer times per day (time/group effect: *p*<0.001)), - Changes in duration at 16-26w (SD): EBF=28 (59), SF=60 (56), SF-M 30 (58) fewer minutes per day (time/group effect: *p*<0.01). - Breast-milk intake (g) remained constant in EBF group but reduced significantly in SF and SF-M groups (time/group effect: *p*<0.001). - Total energy intake was not significantly different between groups at 26 weeks. - Energy from breast milk increased in EBF group by 88kcal per day but decreased in SF and SF-M groups by 306kcal and 126kcal respectively (time/group effect: *p*<0.001) |
| Cohen et al., 1995 | Three-arm randomised trial:  (1) EBF vs (2) introduction of complementary foods at 4m, with *ad lib.* nursing 4-6m, vs (3) introduction of complementary foods at 4m, with maintenance of baseline nursing frequency 4-6m.  Mothers were provided with complementary foods. | At 9 and 12m, groups did not differ (p<0.05) by:   - nursing frequency, - observed food intake at midday meal (g), - percentage of midday meal consumed, - number of foods consumed at midday meal, - usual daily intake - overall food acceptance scores of different food items. |
| Dewey et al., 1999 | Two-arm randomised trial: Both groups received encouragement and motivational messages about breastfeeding.  EBF group instructed to continue EBF to 6m, SF group given complementary foods from 4m to feed twice per day alongside normal breastfeeding | Feeding frequency and total energy were not changed but differences were seen in duration of feeds and total breast milk intake:   - Feeding frequency: No statistically significant changes from baseline to follow-up were found in either SF or EBF group in day time, night time or total breastfeeding frequency. - Total energy intake: No significant differences in total energy intake (kcal) were found between groups at 6m. - Feeding duration: There was a significant change in average time spent feeding per day: decreasing by 38mins in SF group and increasing by 11mins in EBF group (*p*<0.01). - Breast milk intake: SF group average breast milk intake decreased by 39 g/d but increased by 28 g/d in the EBF group (26w intake adjusted for initial breast-milk intake: *p*=0.006) and was significantly different between groups. |
| Flax et al., 2015 | Cluster randomised controlled trial: Participants in both groups received food vouchers and monthly nutrition education. Intervention group also received Plumpy'doz (lipid-based nutrient supplement) | Intervention group had higher macronutrient and micronutrient intakes at endline than control group and the adjusted difference in daily mean intakes between groups at endline (Ediff) were significantly greater than they had been at baseline (Bdiff):   - Total energy (kcal): Bdiff=-0.23, Ediff=0.34, *p*<0.01; - vitamin A (μg): Bdiff=-0.22, Ediff=0.63, *p*<0.001; - vitamin B_12_ (μg): Bdiff=-0.30, Ediff=0.45, *p*<0.05; - folate (μg): Bdiff=-0.30, Ediff=0.72, *p*<0.001; - iron (mg) Bdiff=-0.27, Ediff=0.83, *p*<0.001; - zinc (mg): Bdiff=-0.24, Ediff=0.96, *p*<0.001 |
| Horton et al., 1996 | Secondary data analysis of a cohort study: Breastfeeding promotion programmes conducted at maternity services including education and support | Percentage of children EBF was lower in control (22.2%) than programme (42.7%) groups. |
| Lutter et al., 1994 | Impact evaluation using prospective cohort study: Baby-Friendly Hospital Initiative-related activities | Intervention components significantly associated with EBF at 1m and/or 2m (*p*<0.05) include:   - Demonstration on breast-milk expression - breastfeeding talk, - information on engorgement, - information on sore nipples, - information on sufficient milk (resulting in 7 days longer EBF), - information on increasing milk supply, - information on post-partum breastfeeding help, - information on introduction of liquids.   However, evidence for these associations was not always consistent across the follow up points or across hospitals and sometimes resulted in negative or null associations. |
| Smith, 2002 | Repeated cross-section quasi-experiment: Project HOPE's "Village Health Banks" program of credit only banks with health education vs without health education | No evidence that health banks or credit-only banks increased or decreased the incidence of breastfeeding (*p*>0.2) |
| **El Salvador (*n*=1)** | | |
| Pérez-Escamilla, 2004 | Repeated cross section at baseline and follow-up: Training of healthcare workers and information management system to improve breastfeeding counselling. | Between baseline and the fourth follow-up: % babies breastfed in the delivery room increased from 66% to 72% (*p*<0.001) and % of babies breastfed within the first 30mins increased from 57% to 70% (*p*<0.001) |
| **Policy evaluations** | | |
| **Guatemala (*n*=1)** | | |
| Grajeda et al., 2016 | Mixed methods evaluation of document analysis and cross-sectional survey: *La Estrategia de Comunicación para el Desarrollo en Seguridad Alimentaria y Nutricional:* promoting child feeding practices using communications including socio-dramas, community nutrition lotteries, TV, and radio programmes | Reported knowledge of optimal EBF and complementary feeding practices increased due to project activities however practice lagged due cultural traditions, beliefs and inadequacy of living conditions (specific data not reported) |
| **Honduras (*n*=6)** | | |
| American Public Health Association, 1987 | Pilot study evaluation using repeated cross-sections  *Proyecto de Apoyo a la Lactancia Materna* (PROALMA). Promoting breastfeeding practices by training healthcare professionals to provide counselling to women and changing hospital policies between 1982-1988 | - Any initiation of breastfeeding was similar: 1982=96%; 1985=97% - Average age of stopping breastfeeding increased: 1982=5m; 1985= >1 year - % children breastfed at 12m increased: 1982=35% 1985=70% - Average age of introduction of supplementary bottles increased: 1982=2wks 1985=2.5m - % women introducing supplementary foods at 1m decreased: 1982=65% 1985=40% |
| Canahuati, 1990 | Extension activities evaluation using non-randomised trial (talks+postnatal appointments)  *Proyecto de Apoyo a la Lactancia Materna* (PROALMA). Promoting breastfeeding practices by training healthcare professionals to provide counselling to women and changing hospital policies between 1982-1988 | - Prevalence of any breastfeeding at 6m increased: Control=58.3% Intervention=73.4% (*p*<0.05) - Prevalence of EBF at 90 days increased: Control=13.9% Intervention=23.1% (*p*<0.05) |
| Popkin et al., 1991 | Programme evaluation with repeated cross-section: *Proyecto de Apoyo a la Lactancia Materna* (PROALMA). Promoting breastfeeding practices by training healthcare professionals to provide counselling to women and changing hospital policies between 1982-1988 | Despite projected declines in women initiating breastfeeding due to secular trends, an increase of 2 percentage points between 1981-84 was seen, which authors indicate could be a result of PROALMA (*Proyecto de Apoyo a la Lactancia Materna*) activities |
| Schaetzel et al., 2008 | AIN-C (Atención Integral a la Niñez en la Comunidad) Programme led by the Ministry of Health delivering growth monitoring and promotion activities through community volunteers   Final report: original evaluation plan for pre/post testing of designated AIN-C and control communities was not possible due to a failure of community matching and extensive contamination of control communities with AIN-C activities. A cross-section of participants was therefore randomly sampled (from 92 communities) based on individual-level participation in program activities (AIN) vs no exposure to growth monitoring and promotion (no-GMP) | Key IYCF practices were improved amongst AIN participants compared to controls, but differences were similar to midterm levels   AIN participants were more likely to demonstrate positive feeding behaviours than no-GMP for the following indicators:   - Higher levels of EBF at 3m (68.3% vs 56.7%, no *p*-value reported but described as non-significant), and 6m (55.8% vs 40.0%, no *p*-value reported but described as statistically significant). - Greater median duration of any breastfeeding and EBF (20.9m vs 19.5m and 4m vs 2.4m respectively, no *p*-values reported) - Slower rate of cessation of any breastfeeding and EBF (Log-rank statistic=15.94 (*p*=0.0001); Breslow’s Test=12.90 (*p*=0.0001); Log-rank statistic=4.22 (*p*=0.0399); Breslow’s Test=9.43 (*p*=0.0021)) - Higher % children that met recommendations for breastfeeding and frequency of feeding (38% vs 24.9%, *p*<0.001) - Higher % of children meeting recommendations for feeding frequency and quantity (2.8% vs 0.8%, *p*<0.05)* - Greater proportion of mothers increased breastfeeding during child illness: Diarrhea (45% vs 34.6%, *p*<0.05), Acute respiratory infection (no percentages reported, *p*<0.01) - Greater proportion of mothers increased quantity and quality of other foods during child illness: Diarrhea: (increase quantity=10.6% vs 3.9%, *p*<0.01; increase quality=12.7% vs 4.9%, *p*<0.01); Acute Respiratory Infection ( (increase quantity=4.9% vs 0.7%, *p*<0.05; increase quality=8.5% vs 0.7%, *p*<0.01) but roughly half of mothers in both groups either maintained or increased quantity and quality (data not reported)   *percentages remain very low in both groups |
| Sierra et al., 2019 | AIN-C (Atención Integral a la Niñez en la Comunidad) Programme led by the Ministry of Health delivering growth monitoring and promotion activities through community volunteers: special project of AIN-C implemented by decentralized providers in 1038 rural communities launched in 2008  Quasi-experiment using repeated surveys of intervention and control communities | EBF increased by 4.2% in AIN-C communities compared to controls (no statistical test reported) |
| Van Roekel et al., 2002 | AIN-C (Atención Integral a la Niñez en la Comunidad) Programme led by the Ministry of Health delivering growth monitoring and promotion activities through community volunteers  Mid-term evaluation using community-level longitudinal approach by randomly sampling individuals from sub-set of same community clusters as in baseline | Knowledge of IYCF and some key practices were substantially improved in AIN-C communities compared to control  AIN-C and control communities had similar:   - Percentage of children ever breastfed (96% vs 97%) - Percentage current breastfeeding (75% vs 70%) - Mean daily breastfeeding frequency (0-5m: 10.6 vs 9.2; 6-8m: 11.0 vs 9.0; 9-11m: 9.9 vs 9.4) but the percentage of children fed 13 times or more was consistently higher in AIN-C (0-5m: 37% vs 32%; 6-8m: 55% vs 29%; 9-11m: 38% vs 30%) - Mean age of introducing tea and coffee (4.5 vs 3.7) - Mean age of introducing solid food (5.4 vs 4.8)   Improvements in AIN-C compared with control in:   - Overall knowledge, attitudes and practices score on a scale of 1-9 (6.02 vs 4.91, p≤0.001) - EBF <4m (56% vs 24%; p≤0.001) - EBF <6m (46% vs 19%; p≤0.001) - Higher mean age of introducing water (3.6m vs 2.8m p≤0.01) - Higher mean age of introducing juice (4.7m vs 4.0 p≤0.001) - Higher child feeding scores for children at 6-12 out of 7, (no data reported, p≤0.001) and 12-23m out of 5 (3.02 vs 2.40, p≤0.001).   AIN-C communities were less likely to have introduced the following items by age 6m:   - Water (67% vs 80%; p≤0.001)* - Other milks (43% vs 60%; p≤0.001) - Juice (49% vs 66%; p≤0.001) - Tea or coffee (45%. vs 55%; p<0.01) - Solid foods (40% vs 59%; p≤0.001)   * but difference also significant at baseline |

Notes: AIN-C=Atención Integral a la Niñez en la Comunidad Programme; BCC=behaviour change communication, CSB=corn-soy blend; EBF=exclusive breastfeeding; EIBF=early introduction of breastfeeding; FFR=full family ration; GMP=growth monitoring and promotion; IYCF=infant and young child feeding; LNS=lipid-based micronutrient supplement; m=months; MNP=micronutrient powder; NFR=no family ration; pp=percentage points; PROALMA=Proyecto de Apoyo a la Lactancia Materna; PROCOMIDA= Programa Comunitario Materno Infantil de Diversificación Alimentaria; RFR=reduced family ration; RR=risk ratio; SF=group of children given supplementary food at 4 months; wks=weeks.
